# Involvement of Innate Immune Receptors in the Resolution of Acute Hepatitis B in Woodchucks

**DOI:** 10.3389/fimmu.2021.713420

**Published:** 2021-07-22

**Authors:** Manasa Suresh, Bin Li, Marta G. Murreddu, Severin O. Gudima, Stephan Menne

**Affiliations:** ^1^ Department of Microbiology & Immunology, Georgetown University Medical Center, Washington, DC, United States; ^2^ Department of Microbiology, Molecular Genetics & Immunology, University of Kansas Medical Center, Kansas City, KS, United States

**Keywords:** pattern recognition receptors, hepatitis B virus, acute hepatitis B, innate immune response, woodchuck, chronic hepatitis B

## Abstract

The antiviral property of small agonist compounds activating pattern recognition receptors (PRRs), including toll-like and RIG-I receptors, have been preclinically evaluated and are currently tested in clinical trials against chronic hepatitis B (CHB). The involvement of other PRRs in modulating hepatitis B virus infection is less known. Thus, woodchucks with resolving acute hepatitis B (AHB) after infection with woodchuck hepatitis virus (WHV) were characterized as animals with normal or delayed resolution based on their kinetics of viremia and antigenemia, and the presence and expression of various PRRs were determined in both outcomes. While PRR expression was unchanged immediately after infection, most receptors were strongly upregulated during resolution in liver but not in blood. Besides well-known PRRs, including TLR7/8/9 and RIG-I, other less-characterized receptors, such as IFI16, ZBP1/DAI, AIM2, and NLRP3, displayed comparable or even higher expression. Compared to normal resolution, a 3–4-week lag in peak receptor expression and WHV-specific B- and T-cell responses were noted during delayed resolution. This suggested that PRR upregulation in woodchuck liver occurs when the mounting WHV replication reaches a certain level, and that multiple receptors are involved in the subsequent induction of antiviral immune responses. Liver enzyme elevations occurred early during normal resolution, indicating a faster induction of cytolytic mechanisms than in delayed resolution, and correlated with an increased expression of NK-cell and CD8 markers and cytolytic effector molecules. The peak liver enzyme level, however, was lower during delayed resolution, but hepatic inflammation was more pronounced and associated with a higher expression of cytolytic markers. Further comparison of PRR expression revealed that most receptors were significantly reduced in woodchucks with established and progressing CHB, and several RNA sensors more so than DNA sensors. This correlated with a lower expression of receptor adaptor and effector molecules, suggesting that persistent, high-level WHV replication interferes with PRR activation and is associated with a diminished antiviral immunity based on the reduced expression of immune cell markers, and absent WHV-specific B- and T-cell responses. Overall, the differential expression of PRRs during resolution and persistence of WHV infection emphasizes their importance in the ultimate viral control during AHB that is impaired during CHB.

## Introduction

The host innate immunity recognizes viral infections and activates appropriate defense mechanisms. An important part of this response is mediated by germline-encoded receptors, also called pathogen recognition receptors (PRRs) that bind pathogen-associated molecular patterns (PAMPs), ranging from viral nucleic acids to their encoded glycoproteins (1). Subsequent activation of PRRs triggers their downstream signaling pathways, involving adaptor molecules, transcription factors, and type-I interferons (IFNs) or pro-inflammatory cytokines, leading to cell intrinsic and antiviral immune responses (2). The known PRR families include toll-like receptors (TLRs), retinoic acid-inducible gene I (RIG-I) like receptors (RLRs), nucleotide-binding oligomerization domain (NOD) like receptors (NLRs), cytosolic DNA sensors (CDSs), and inflammasomes. The subcellular localization of these PRRs in immune and/or non-immune cells is either on plasma and endosomal membranes or within the cytoplasm and nucleus.

Hepatitis B virus (HBV) is a serious global health issue, with 257 million chronic carriers who are at high risk of developing chronic hepatitis B (CHB), liver cirrhosis, and hepatocellular carcinoma (3, July 8). HBV infection in adults usually leads to acute hepatitis B (AHB) and is often self-limited *via* the control by a strong antiviral immune response, with only a 5% chance of progressing to chronicity (4). However, HBV infection acquired at birth by mother-to-child transfer results in CHB in 95% of cases (5). CHB is associated with an inadequate antiviral immune response, which is responsible for the progression of liver disease. The surge in and the continuous presence of viral proteins during HBV replication interferes with many functions of the innate and adaptive immunity (6). These immunodeficiencies present in CHB have shifted the focus of drug development to immunomodulation as a therapeutic strategy for reviving the impaired immune response in patients with chronic HBV infection (7). The underlying reason is that current treatment options for CHB, including nucleos(t)ide analogs and (pegylated) IFN-α, rarely lead to immunological control of HBV infection (i.e., functional cure) and are further associated with adverse effects and viral relapse upon treatment discontinuation.

Small agonist molecules targeting selected PRRs have been recently developed, with several compounds tested in preclinical animal models of HBV and subsequently in patients with CHB (8). These includes compounds activating TLR3, TLR7, TLR8, TLR9, stimulator of interferon genes (STING), and RIG-I. In woodchucks infected with the HBV-like woodchuck hepatitis virus (WHV), several of these agonist molecules suppressed viral replication and mediated sustained antiviral effects. These studies have highlighted the important role of innate immune receptors in facilitating a coordinated adaptive immune response and hence are suitable targets for immune modulation.

Besides the above PRRs, the involvement of other viral nucleic acid sensing receptors in HBV infection, and especially in AHB resolution, remains unknown. Main reasons are that studying the early phase of HBV infection in patients is challenging due to its asymptomatic nature and that obtaining frequent liver biopsies from patients is limited. The Eastern woodchuck (*Marmota monax*) infected with WHV is a fully immunocompetent animal model that has been extensively used for investigating HBV-associated pathogenesis during the resolved *versus* chronic outcome of infection. Similar to HBV infection in humans, almost all adult woodchucks resolve WHV infection, whereas neonatal woodchucks infected shortly after birth mainly progress to CHB (9). Thus, woodchucks are an excellent animal model for studying the early events of host immune response to the virus following experimental WHV infection and for testing the safety and antiviral efficacy of novel drugs for the treatment of CHB. In our previous work on acute, self-limited WHV infection in adult woodchucks, we found that the coordinated interplay of innate and adaptive immune responses mediates resolution of AHB, with an initial viral control by natural killer- (NK-) cell associated IFN-γ production (10). In the present study, we investigated the kinetics of viral nucleic acid sensing PRRs in the liver and periphery of adult woodchucks with resolving WHV infection. Comparing the intrahepatic peak upregulation of important receptors during AHB to their expression and presence in the setting of established and progressing CHB revealed an involvement of viral DNA sensors in the control of WHV infection, and much more so than viral RNA sensors.

## Materials and Methods

### Ethics Statement

The animal protocol entitled “Super-infection and virus spread during chronic hepadnaviral infection” and all procedures involving woodchucks were approved by the IACUC of Northeastern Wildlife, Inc. (Harrison, ID) on January 3, 2012, and adhered to the national guidelines of the NIH Guide for the Care and Use of Laboratory Animals. Woodchuck were anesthetized by intramuscular injection of ketamine (50 mg/kg) and xylazine (5 mg/kg) for blood collection and percutaneous liver biopsy. Prior to euthanasia, woodchucks were anesthetized as described above and euthanized by an overdose of pentobarbital (80-100 mg/kg) administered by intracardiac injection, followed by bilateral intercostal thoracotomy.

### Woodchucks and WHV Parameters

The ten adult woodchucks investigated were part of an animal cohort that was experimentally infected with an equal number of genome equivalents (GE) of WHV (11, 12). Animals were selected based on the duration of AHB resolution that allowed studying PRR expression in detail during the early stages of WHV infection. Woodchucks were further characterized as animals with normal or delayed resolution based on the individual kinetics of viremia and antigenemia in serum and liver. Following WHV inoculation, all woodchucks were monitored for approximately 14 to 18 weeks. Serum, blood, and liver samples were collected prior to inoculation (pre-inoculation or Pre) and again at the end of the study (EOS). In addition, serum was collected weekly, blood was obtained weekly or biweekly, and additional liver biopsies were taken at weeks +5/6, +9, and +12/13. Serum viral load, intrahepatic WHV replication, and innate and adaptive immune responses in some of these animals were reported recently (10–12). For woodchucks with established CHB, viremia and antigenemia in serum and liver, as well as antibody and T-cell responses in the periphery, were determined as described for animals resolving AHB. Liver tissues were further examined for sinusoidal and portal hepatitis and a composite score was obtained on a scale of 0-6, where 0 indicated absent, >0-2 indicated mild, >2-4 indicated moderate, and >4 indicated severe/marked inflammation.

### Intrahepatic and Peripheral PRR Expression

Total RNA from liver biopsy samples was isolated using the RNeasy Mini kit (Qiagen, Redwood City, CA) by following the manufacture’s protocol. Total RNA from whole blood collected in PAXgene blood tubes (Qiagen) was isolated using the PAXgene Blood miRNA kit (Qiagen) with on-column DNase I digestion using RNase-free DNase according to the manufacturer’s instructions. RNA concentrations were measured using a Nano Drop 8000 spectrophotometer (Thermo Scientific, Waltham, MA). Liver and blood derived mRNA samples were then reverse transcribed using random primers and the High Capacity cDNA Reverse Transcription kit (Applied Biosystems, Foster City, CA). Changes in the transcript level of various PRRs, adaptor molecules, transcription factors, and effector molecules and cytokines (**Supplementary Table S1**) in the liver and blood during the course of acute WHV infection were determined using real-time PCR and woodchuck-specific primers and probes (**Supplementary Table S2**), as described previously (10). A fold-change of ≥2.1 from the pre-inoculation baseline was considered a positive result for increased molecule expression. Expression results for the above molecules in woodchuck liver are provided in **Supplementary Tables S3** and **S4**.

### Immunohistochemistry

Liver tissues were stained with cross-reactive antibodies to selected PRRs, including RIG-I (Origene Technologies, Rockville, MD), TLR7 (Abcam, Cambridge, MA), TLR8 (Bioss, Woburn, MA), ZBP1 (Bioss), and NLRP3 (Bioss). Whole slide images were obtained using an Aperio Scanner GT450 (Leica Biosystems, Buffalo Grove, IL) and analyzed by QuPath, a quantitative pathology and bioimaging software developed at the University of Edinburg, UK (13). Five random areas of similar size were selected in each of the images and a set of same parameters was applied to each area for the detection of positively stained cells. The output results included the total number of immune and non-immune cells detected and the number of cells that stained positively. The percentage of stained cells in each whole slide image is presented as an average of the five areas. The number of positively stained immune cells in the same five areas was obtained by manual counting, a percentage was calculated based on the total number of cells detected and then averaged.

### PRR Presence in Woodchucks With AHB and CHB and in WHV-Uninfected Controls

For comparing PRR expression during the acute and chronic outcomes of WHV infection, the following sample sets were used: (i) the pre-inoculation and peak expression levels of each receptor in the liver of the ten woodchucks with resolving WHV infection served as samples for the uninfected control or peak AHB, respectively; and (ii) receptor expression in historical liver tissues of ten age- and gender-matched, treatment-naïve, established chronic WHV carrier woodchucks were used as samples for progressing CHB. For each sample set, the PRR expression was averaged, and the percentage of increase or decrease in receptor transcript level during AHB and CHB was calculated relative to the uninfected control. A similar comparison was performed for innate and adaptive immune cell markers in each sample set. Furthermore, for comparing PRR presence at the protein level, liver tissues from each sample set were stained for selected receptors using the above-described cross-reactive antibodies.

### Statistical Analysis

Statistical comparisons were performed using unpaired Student’s *t*-test with equal variance for the intrahepatic expression of PRRs, adaptor molecules, interferon-stimulated genes (ISGs), and immune cell markers between woodchucks with AHB and CHB.

## Results

### Course of WHV Infection in Woodchucks

**Figure 1:**
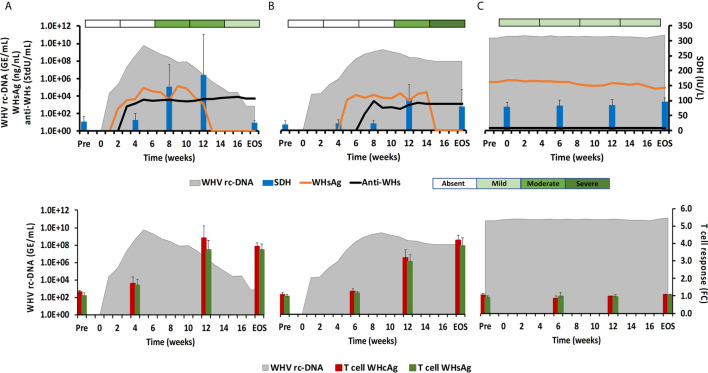
Course of WHV infection in the periphery. Kinetics of serum viremia [WHV rc-DNA, as already reported (11, 12)] and antigenemia (WHsAg), antibody response (anti-WHs antibodies), liver enzyme (SDH) activity, and liver inflammation (top panels) and of blood T-cell response (specific to WHcAg and WHsAg) with WHV rc-DNA kinetics (bottom panels) in woodchucks during **(A)** AHB with normal resolution (n=5), **(B)** AHB with delayed resolution (n=5), and **(C)** CHB progression (n=5). Changes in WHV rc-DNA, WHsAg, and anti-WHs antibodies are plotted on the left y-axes, while changes in SDH and WHcAg- and WHsAg-specific T-cell responses are plotted on the right y-axes. Horizontal bars represent the standard error of the mean. The composite score for sinusoidal and portal hepatitis is represented as a scale bar, indicating absent, mild, moderate, or severe/marked liver inflammation. GE, genomic equivalents; IU, international unit; FC, fold-change in T-cell proliferation from unstimulated controls; Pre, pre-inoculation for AHB and start of study for CHB; EOS, end of study.

**Figure 2:**
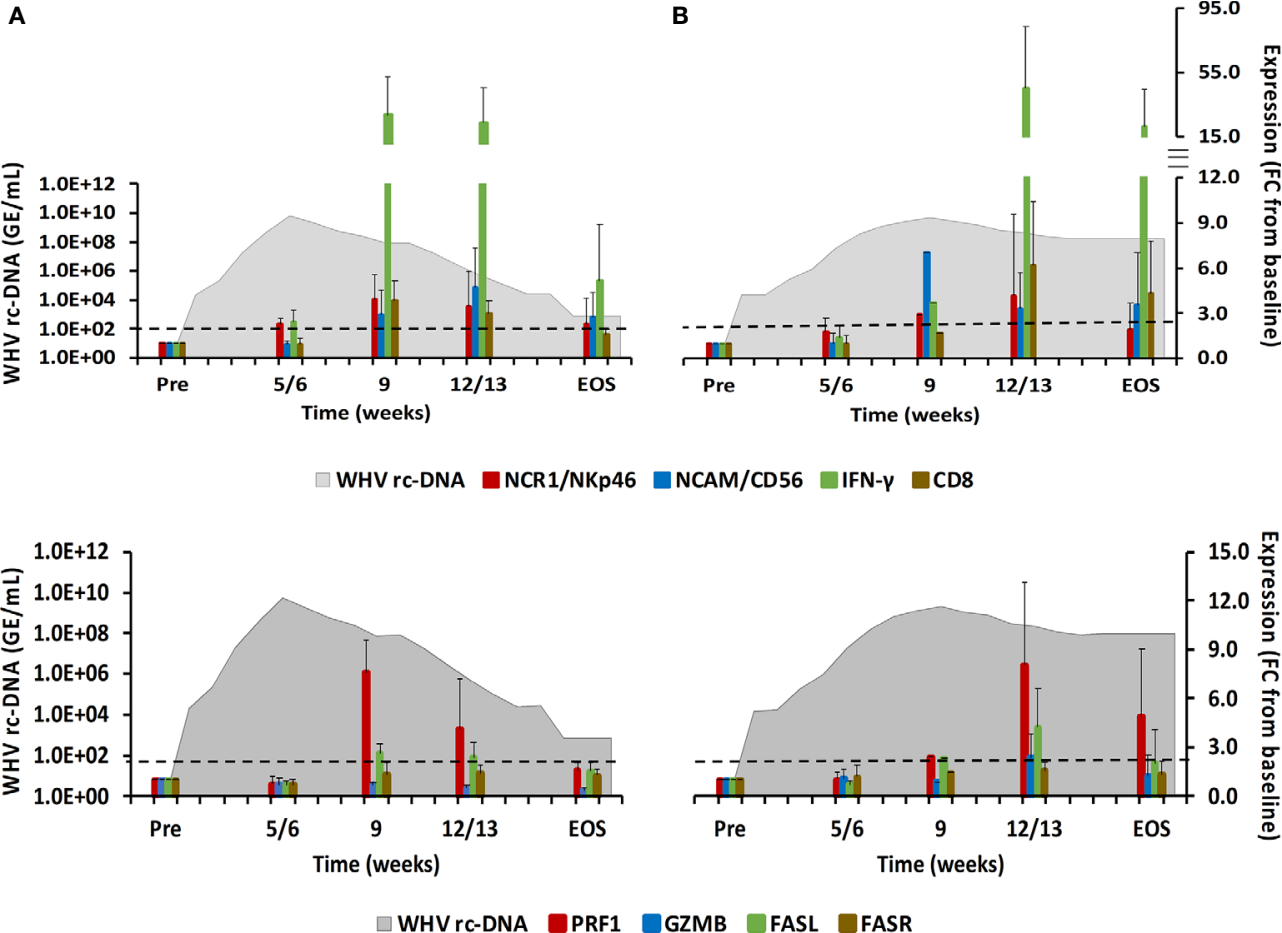
Intrahepatic immune cell marker and cytolytic effector molecule expression. Expression changes of NK-cell and CTL markers (top-panels) and of the cytolytic effector molecules PRF, GZMB, FASL, and FASR (bottom panels) in liver with WHV rc-DNA kinetics of woodchucks during AHB with **(A)** normal resolution (n=5) and **(B)** delayed resolution (n=5). The fold-changes in immune cell marker and cytolytic effector molecule transcript level is plotted on the right y-axis, while serum WHV rc-DNA loads are plotted on the left y-axis. The horizontal, dotted line indicates the cutoff for positive expression (i.e., ≥2.1-fold increase from the pre-inoculation baseline). Horizontal bars represent the standard error of the mean. Pre, pre-inoculation for AHB; EOS, end of study; FC, fold-change.

The ten adult woodchucks investigated in this study were part of an animal cohort that was inoculated with WHV and then followed for 14 to 18 weeks (11, 12). The course of acute WHV infection in individual animals has been described previously (10–12), including serum viremia, antigenemia, antibody response, intrahepatic WHV replication and antigen expression, and liver enzyme levels. Overall, the increases and subsequent declines of the above infection parameters indicated AHB in all animals, which eventually resolved. Based on the kinetics of viremia and antigenemia, these adult woodchucks were separated into animals with normal or delayed resolution, with five animals in each group. For comparison with progressing chronic infection, serum, blood, and liver samples obtained from five woodchucks with established CHB over a period of 18 weeks were included. The average changes in WHV relaxed circular (rc) DNA and surface antigen (WHsAg) loads, anti-WHsAg antibody (anti-WHs) titer, sorbitol dehydrogenase (SDH) liver enzyme level, and liver inflammation are provided in **Figure 1** for depicting differences in the course of AHB between woodchucks with normal and delayed resolution, as well as animals with CHB progression. For comparing the immune responses associated with liver inflammation in the setting of normal and delayed AHB resolution, the intrahepatic expression kinetics of NK-cell and cytotoxic CD8+ T-cell (CTL) markers and of cytolytic effector molecules are shown in **Figure 2**. These included natural cytotoxicity triggering receptor 1 (NCR1/NKp46), neural cell adhesion molecule (NCAM/CD56), IFN-γ, cluster of differentiation (CD) 8, perforin (PRF), granzyme B (GZMB), and apoptosis-inducing FAS ligand (FASL) and receptor (FASR). The changes in WHV rc-DNA levels are further used in the following figures for correlating the expression of PRRs and other immune response markers with acute and chronic WHV infection.

During normal resolution (**Figure 1**), the peak in serum WHV rc-DNA in woodchucks was observed at week 5. WHsAg and anti-WHs antibodies were detectable as early as weeks 2 or 3, respectively. The SDH level increased at week 8 and was maximal at week 12, which coincided with moderate liver inflammation and the peak in WHcAg- and WHsAg-specific T-cell responses. The peak expression of NK-cell and CTL markers (fold-change: NKp46, 4.0; NCAM, 2.9; IFN-γ, 34.1; CD8, 3.9) and of cytolytic effector molecules (fold-change: PRF, 7.6; FASL, 2.6) was almost always observed at week 9 (**Figure 2**). The average expression of GZMB and FASR revealed no increases that were considered positive (i.e., ≥2.1-fold from the pre-inoculation baseline). In animals with delayed resolution (**Figure 1**), there was a 3–4-week lag in the peak serum viremia and in the detection of antigenemia and antibodies (WHV rc-DNA at week 9, WHsAg at week 5 and anti-WHs at week 7). Similarly, the increase and peak in SDH level was observed later at week 12, with concentrations lower than during normal resolution. Liver inflammation, however, was often more pronounced in individual animals, ranging from moderate to severe hepatitis between week 12/13 and the EOS. This was further associated with a higher expression of NK-cell and CTL markers (fold-change: NKp46, 4.2; NCAM, 7.1; IFN-γ, 45.1; CD8, 6.2) and cytolytic effector molecules (fold-change: PRF, 8.0; GZMB, 2.4; FASL, 4.2) at week 12/13, except for NCAM with a maximum at week 9 (**Figure 2**). FASR expression again remained close to the baseline. Furthermore, the peak in WHV-specific T-cell responses was also noted later at week 18. In contrast to woodchucks with AHB, animals with established and progressing CHB had nearly unchanged serum WHV rc-DNA and WHsAg levels at high concentrations during the 18-week period, with undetectable anti-WHs antibodies, rather uniform SDH levels, mild liver inflammation, and absent WHV-specific T-cell responses.

### Intrahepatic PRR Presence During AHB Resolution and CHB Progression

PRRs sensing viral nucleic acids and then triggering downstream signaling pathways for the induction of an antiviral immune response include diverse families of receptors, such as RLRs, NLRs, TLRs, CDSs, and inflammasomes. For investigating the presence and upregulation of these PRRs during resolution of acute WHV infection or progression of chronic WHV infection, their expression in woodchuck liver and blood was determined prior to and following WHV inoculation (14). Complete liver data sets are presented from all ten woodchucks with AHB, and nearly complete blood data sets are available from three woodchucks each with normal or delayed resolution. Intrahepatic expression of PRRs in three animals with progressing CHB was determined as well but remained close to the baseline and did not show pronounced changes for most receptors. Blood expression of PRRs in animals with CHB was not determined.

#### RLRs

**Figure 3:**
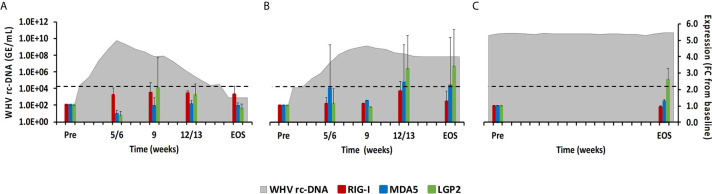
Intrahepatic RLR expression. Expression changes of RIG-I, MDA5, and LGP2 in liver with WHV rc-DNA kinetics of woodchucks during **(A)** AHB with normal resolution (n=5), **(B)** AHB with delayed resolution (n=5), and **(C)** CHB progression (n=3). The fold-changes in receptor transcript level are plotted on the right y-axis, while serum WHV rc-DNA loads are plotted on the left y-axis. The horizontal, dotted line indicates the cutoff for positive expression (i.e., ≥2.1-fold increase from the pre-inoculation baseline). Horizontal bars represent the standard error of the mean. Pre, pre-inoculation for AHB and start of study for CHB; EOS, end of study; FC, fold-change.

This PRR family includes RIG-I, melanoma differentiation-associated protein 5 (MDA5), and laboratory of genetics and physiology 2 (LGP2), which are mainly expressed in the cytosol of non-immune cells, including hepatocytes, but are sometimes also expressed in immune cells, and that sense viral single-stranded (ss) RNA (RIG-I and MDA5) or double-stranded (ds) RNA (LGP2) (15). Upon binding of their PAMP, the caspase recruitment domain (CARD) of RIG-I and MDA5 interacts with the CARD domain of the adaptor molecule, mitochondrial antiviral signaling protein (MAVS). This interaction then activates the downstream signaling pathway involving nuclear factor kappa-light-chain-enhancer of activated B-cells (NF-κB) and IFN-regulating factor (IRF) 3 and 7 as transcription factors to produce type-I IFNs, especially IFN-α (16, 17). LGP2 is a less-characterized receptor and is mainly considered a negative regulator of RIG-I and MDA5. In woodchuck liver (**Figure 3**), the average RIG-I expression during normal and delayed resolution revealed no increases over time. MDA5 and LGP2 expression also remained unchanged during normal resolution but was increased and/or peaked between week 12/13 and the EOS during delayed resolution (fold-change: MDA5, 2.4; LGP2, 3.4). LGP2 was the only receptor in animals with progressing CHB with somewhat increased expression at the EOS (fold-change: LGP2, 2.6). In woodchuck blood (**Supplementary Figure 1**), increased RLR expression was observed for LGP2 at week 12 during delayed resolution (fold-change: 2.4), but not for RIG-1 and MDA5.

#### NLRs

**Figure 4:**
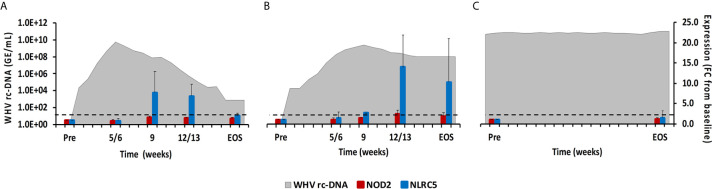
Intrahepatic NLR expression. Expression changes of NOD2 and NLRC5 in liver with WHV rc-DNA kinetics of woodchucks during **(A)** AHB with normal resolution (n=5), **(B)** AHB with delayed resolution (n=5), and **(C)** CHB progression (n=3). The fold-changes in receptor transcript level are plotted on the right y-axis, while serum WHV rc-DNA loads are plotted on the left y-axis. The horizontal, dotted line indicates the cutoff for positive expression (i.e., ≥2.1-fold increase from the pre-inoculation baseline). Horizontal bars represent the standard error of the mean. Pre, pre-inoculation for AHB and start of study for CHB; EOS, end of study; FC, fold-change.

This PRR family is characterized by a tandem CARD domain at the amino-terminal end, followed by the conserved NOD domain and leucine-rich repeat (LRR) motifs at the carboxy-terminal end that bind PAMPs (18, 19). Most NLRs are located within the cytosol of immune and non-immune cells, except for NOD-like receptor family CARD domain containing 5 (NLRC5) that also translocates into the nucleus (18). Family members include NOD2, NLRC5, and NOD-like pyrin domain-containing 3 (NLRP3), which is described later with the inflammasomes. NOD2 senses ssRNA and induces type-I IFNs *via* the MAVS-IRF3 signaling pathway (18). NLRC5 is a receptor inducible by IFN-α and IFN-γ (18). In woodchuck liver (**Figure 4**), an increase in NOD2 expression was only observed during delayed resolution, with a peak expression at week 12/13 (fold-change: 2.4). Maximum NLRC5 expression was noted at week 9 during normal resolution (fold-change: 7.8) and at week 12/13 during delayed resolution (fold-change: 14.0). In woodchuck blood (**Supplementary Figure 2**), NOD2 expression was unchanged in both resolution outcomes. NLRC5 expression was maximal at the EOS during normal resolution (fold-change: 2.2) and at weeks 4 and 12 during delayed resolution (fold-change: 2.5-3.5).

#### TLRs

**Figure 5:**
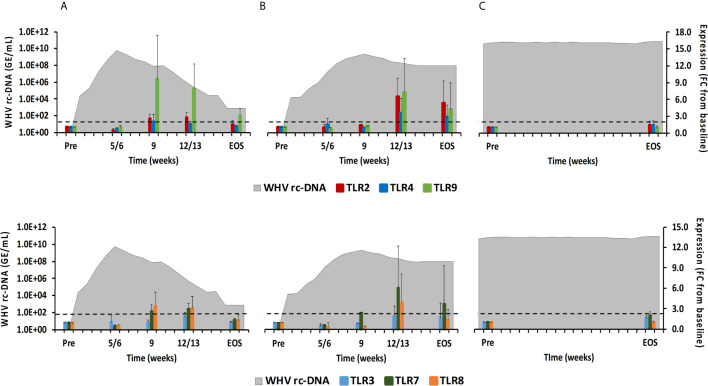
Intrahepatic TLR expression. Expression changes of TLR2/4/9 (top panels) and TLR3/7/8 (bottom panels) in liver with WHV rc-DNA kinetics of woodchucks during **(A)** AHB with normal resolution (n=5), **(B)** AHB with delayed resolution (n=5), and **(C)** CHB progression (n=3). The fold-changes in receptor transcript level are plotted on the right y-axis, while serum WHV rc-DNA loads are plotted on the left y-axis. The horizontal, dotted line indicates the cutoff for positive expression (i.e., ≥2.1-fold increase from the pre-inoculation baseline). Horizontal bars represent the standard error of the mean. Pre, pre-inoculation for AHB and start of study for CHB; EOS, end of study; FC, fold-change.

TLRs have been widely studied in regard to HBV, since their function can be inhibited by viral proteins, but also activated by small agonist molecules for bringing out antiviral effects (8, 20). TLRs are usually expressed in immune cells on the surface or within the endosome (21). TLR2 and TLR4 are expressed on the cell surface and recognize viral proteins. TLR3 (senses ss/dsRNA), TLR7/8 (sense ssRNA), and TLR9 (usually senses unmethylated dsDNA containing cytosine and guanine triphosphate deoxynucleotide (CpG) motifs) are located on endosomal membranes and produce mainly IFN-α *via* the myeloid differentiation primary-response protein 88 (MyD88) adaptor molecule and the IRF3/5/7 downstream pathways (17, 21). In woodchuck liver (**Figure 5**), peak expression of TLR8 and TLR9 was observed at week 9 during normal resolution (fold-change: 3.4 or 9.6, respectively), and at week 12/13 for TLR2 and TLR7 (fold-change:2.7 or 3.0, respectively). Expression of TLR3 and TLR4 remained close to the baseline. During delayed resolution, peak expression of all TLRs, except TLR3, was noted at week 12/13 (fold-change: TLR2, 6.5; TLR4, 3.6; TLR7, 6.2; TLR8, 4.0; and TLR9, 7.2). In woodchuck blood (**Supplementary Figure 3**), expression increases were only observed for TLR3 at the EOS during normal resolution (fold-change: 3.0) and for TLR3 and TLR4 at week 4 and 12, respectively, during delayed resolution (fold-change: TLR3, 2.1; TLR4, 2.6).

#### CDSs

**Figure 6:**
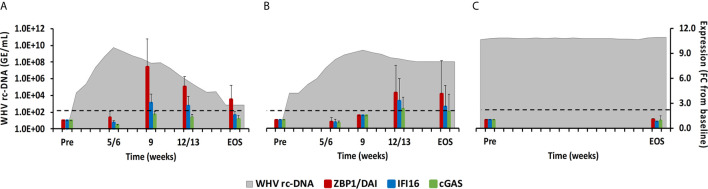
Intrahepatic CDS expression. Expression changes of ZBP1/DA1, IFI16, and cGAS in liver with WHV rc-DNA kinetics of woodchucks during **(A)** AHB with normal resolution (n=5), **(B)** AHB with delayed resolution (n=5), and **(C)** CHB progression (n=3). The fold-changes in receptor transcript level are plotted on the right y-axis, while serum WHV rc-DNA loads are plotted on the left y-axis. The horizontal, dotted line indicates the cutoff for positive expression (i.e., ≥2.1-fold increase from the pre-inoculation baseline). Horizontal bars represent the standard error of the mean. Pre, pre-inoculation for AHB and start of study for CHB; EOS, end of study; FC, fold-change.

Molecules located within the cytosol of immune and non-immune cells have been identified that sense dsDNA and induce type-I IFNs, especially IFN-β (22). The downstream signaling pathway of these PRRs apparently involves STING as the adaptor molecule and TANK-binding kinase 1 (TBK1) and IRF3 for type-I IFN production. Family members include ZBP1/DAI, IFI16, cyclic GMP-AMP synthase (cGAS), DExH-box helicase 9 (DHX9), and DEAH-box helicase 36 (DHX36). In woodchuck liver (**Figure 6**), peak expression of IFI16 and ZBP1/DAI was noted at week 9 during normal resolution (fold-change: 3.1 or 7.4, respectively) and at week 12/13 during delayed resolution (fold-change: 3.3 or 4.2, respectively). An increase in cGAS expression was only observed during delayed resolution at week 12/13 (fold-change: 2.3). DHX9 and DHX36 expression was unchanged in the liver of all woodchucks and in blood of most animals (data not shown). In woodchuck blood (**Supplementary Figure 4**), the expression of IFI16 and ZBP1/DAI was rather unchanged, except for an increase at week 4 during normal resolution (fold-change: ZBP1/DAI, 3.3). cGAS expression in blood was not determined due to insufficient RNA amounts.

#### Inflammasomes

**Figure 7:**
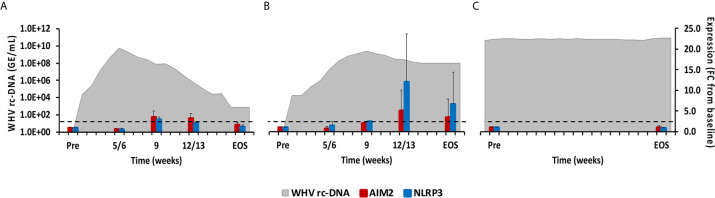
Intrahepatic inflammasome expression. Expression changes of AIM2 and NLRP3 in liver with WHV rc-DNA kinetics of woodchucks during **(A)** AHB with normal resolution (n=5), **(B)** AHB with delayed resolution (n=5), and **(C)** CHB progression (n=3). The fold-changes in receptor transcript level are plotted on the right y-axis, while serum WHV rc-DNA loads are plotted on the left y-axis. The horizontal, dotted line indicates the cutoff for positive expression (i.e., ≥2.1-fold increase from the pre-inoculation baseline). Horizontal bars represent the standard error of the mean. Pre, pre-inoculation for AHB and start of study for CHB; EOS, end of study; FC, fold-change.

Cytosolic nucleic acids produced during viral replication or as damage-associated molecular patterns are sensed by PRRs, which triggers the formation of multiprotein complexes called inflammasomes that are located in the cytoplasm and/or nucleus of immune and non-immune cells (23). Inflammasomes present in liver include Absent in Melanoma 2 (AIM2) and NLRP3. After binding of their PAMP, both inflammasomes interact with apoptosis-associated speck-like protein containing CARD (ASC) as the adaptor molecule. This activates caspase-1 and results in the production of the inflammatory cytokines IL-1β and IL-18 (23). In woodchuck liver (**Figure 7**), peak expression of both inflammasomes was observed at week 9 during normal resolution and at week 12/13 during delayed resolution (fold-change: AIM2, 3.6 or 5.0; NLRP3, 3.3 or 12.0, respectively). In woodchuck blood (**Supplementary Figure 5**), however, inflammasome expression was not apparent, except for NLRP3 at the EOS during normal resolution (fold-change: 2.3).

#### Adaptor Molecules and Transcription Factors

**Figure 8:**
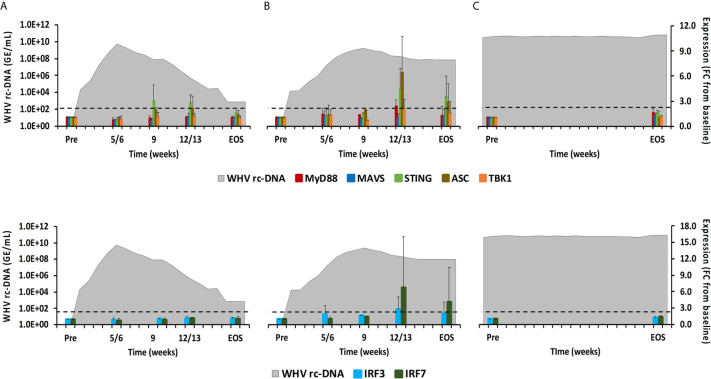
Intrahepatic adaptor molecule and transcription factor expression. Expression changes of adaptor molecules MyD88, MAVS, STING, ASC, and TBK1 (top panels) and transcription factors IRF3 and IRF7 (bottom panels) in liver with WHV rc-DNA kinetics of woodchucks during **(A)** AHB with normal resolution (n=5), **(B)** AHB with delayed resolution (n=5), and **(C)** CHB progression (n=3). The fold-changes in adaptor molecule and transcription factor transcript level are plotted on the right y-axis, while serum WHV rc-DNA loads are plotted on the left y-axis. The horizontal, dotted line indicates the cutoff for positive expression (i.e., ≥2.1-fold increase from the pre-inoculation baseline). Horizontal bars represent the standard error of the mean. Pre, pre-inoculation for AHB and start of study for CHB; EOS, end of study; FC, fold-change.

As described above, PRRs initiate downstream signaling *via* receptor-specific adaptor molecules following PAMP binding, including MyD88, MAVS, STING, TBK1, and ASC (24). This leads to the activation of common transcription factors, such as IRF3/5/7, or the formation of inflammasomes, which ultimately mediates the production of type-I IFNs and pro-inflammatory cytokines (1). In woodchuck liver (**Figure 8**), the expression of MAVS and TBK1 did not increase during resolution. MyD88 and ASC expression remained close to the baseline during normal resolution but showed peak expression at week 12/13 during delayed resolution (fold-change: MyD88, 2.3; ASC, 6.3). The peak in STING expression was noted at weeks 9 or 12/13, respectively, during normal and delayed resolution (fold-change: 3.0 or 4.5, respectively). Expression of IRF3 and IRF7 remained unchanged during normal resolution but was maximal increased at week 12/13 during delayed resolution (fold-change: IRF3, 2.8; IRF7, 6.8). Similar to PRRs, changes in the expression of adaptor molecules and transcription factors were not noted in animals with CHB progression. Expression of adaptor molecules and transcription factors in blood was not tested due to insufficient RNA amounts.

**Figure 9:**
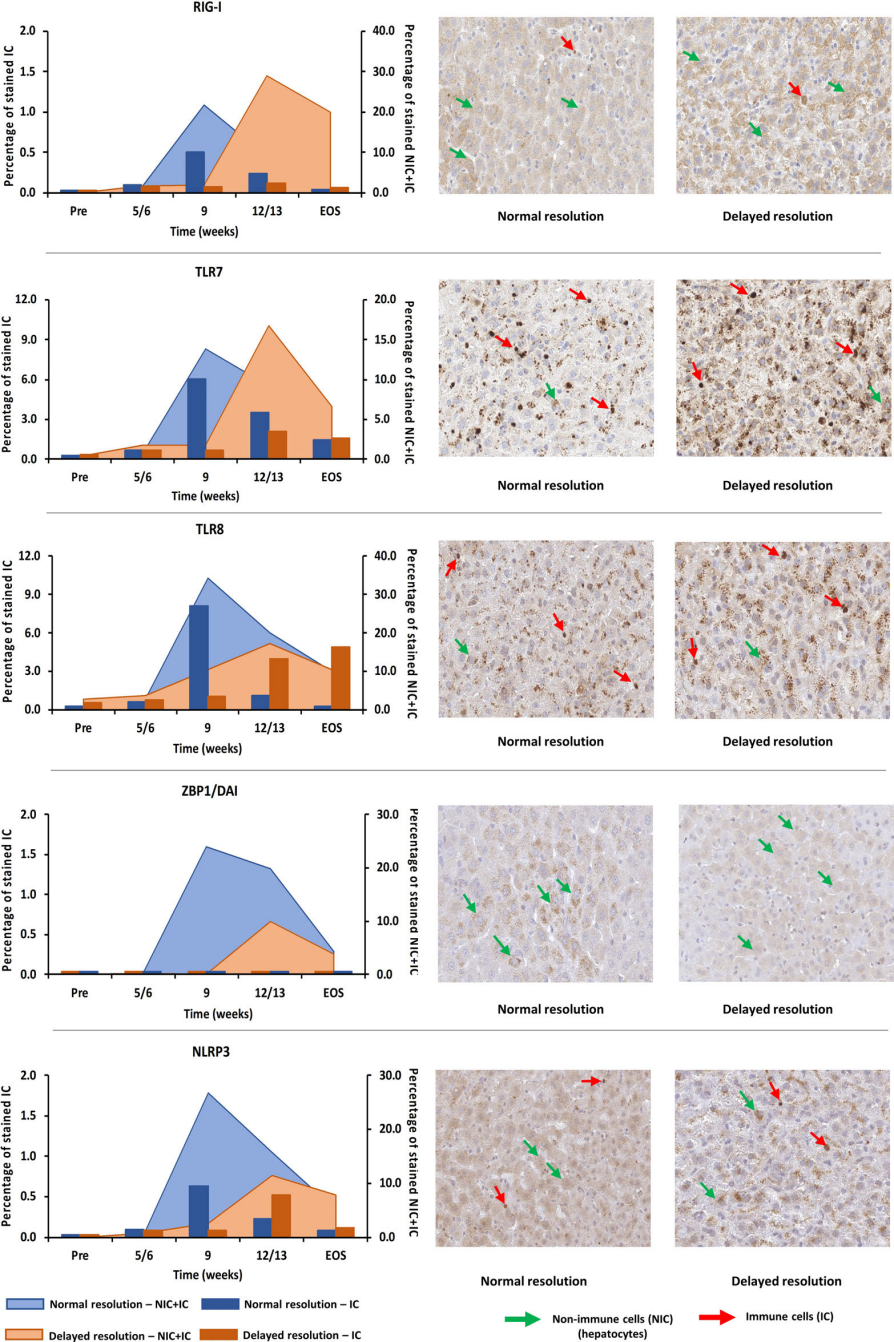
Intrahepatic presence of selected PRRs. Liver tissues from one representative woodchuck each during AHB with normal (M7392) or delayed resolution (M7249) were stained for viral RNA (RIG-I, TLR7, and TLR8) and viral DNA sensing receptors (ZBP1/DAI and NLRP3) using cross-reactive antibodies. The kinetics in the percentage of positively stained cells are presented (left panels). The percentage of stained immune and non-immune cells is plotted on the right y-axis, while the percentage of stained immune cells is plotted on the left y-axis. Representative staining images for the cellular and sub-cellular localization of receptors are shown (right panels). Pre, pre-inoculation; EOS, end of study; NIC, non-immune cells; IC, immune cells.

For verifying the upregulation of selected PRRs during AHB at the protein level, liver tissues from one representative woodchuck with normal (M7392) or delayed resolution (M7249) were stained by immunohistochemistry (IHC) for RIG-I, TLR7, TLR8, ZBP1/DAI, and NLRP3 using cross-reactive antibodies (**Supplementary Figures 6A–E** and **Figure 9**). PRR staining in the liver of both animals was then compared to the respective intrahepatic receptor expression. This revealed that the changes in the percentage of positively stained cells for these receptors followed an almost similar kinetic as observed at the transcript level, with peaks at week 9 during normal resolution (RIG-I, 21.8%; TLR7, 13.8%; TLR8, 34.2%; ZBP1/DAI, 24.0%; NLRP3, 26.7%) and at week 12/13 during delayed resolution (RIG-I, 29.0%; TLR7, 16.7%; TLR8, 17.3%; ZBP1/DAI, 10.0%; NLRP3, 11.4%). Since PRR expression was determined in whole liver biopsy samples, the transcription analysis could not discriminate between cell subsets expressing these receptors. However, based on the IHC results (**Figure 9**), RIG-I, TLR7, TLR8, and NLRP3 were present in both immune and non-immune cells, while ZBP1/DAI was only observed in hepatocytes, but not in monocytes and macrophages. The subcellular localization of RIG-I, ZBP1/DAI and NLRP3 was cytoplasmic, while those of TLR7 and TLR8 was endosomal, based on the more granular staining. The peak in the percentage of infiltrating immune cells positively stained for RIG-I and NLRP3 remained less than 1% of all cells during normal and delayed resolution. Contrary, the peak percentage of immune cells positive for TLR7 and TLR8 staining ranged between 6.1 and 8.1% and between 2.1 and 4.0% of all cells during normal or delayed resolution, respectively.

### Differential Intrahepatic Presence of PRRs and Immune Cells Markers During AHB Resolution and CHB Progression

**Figure 10:**
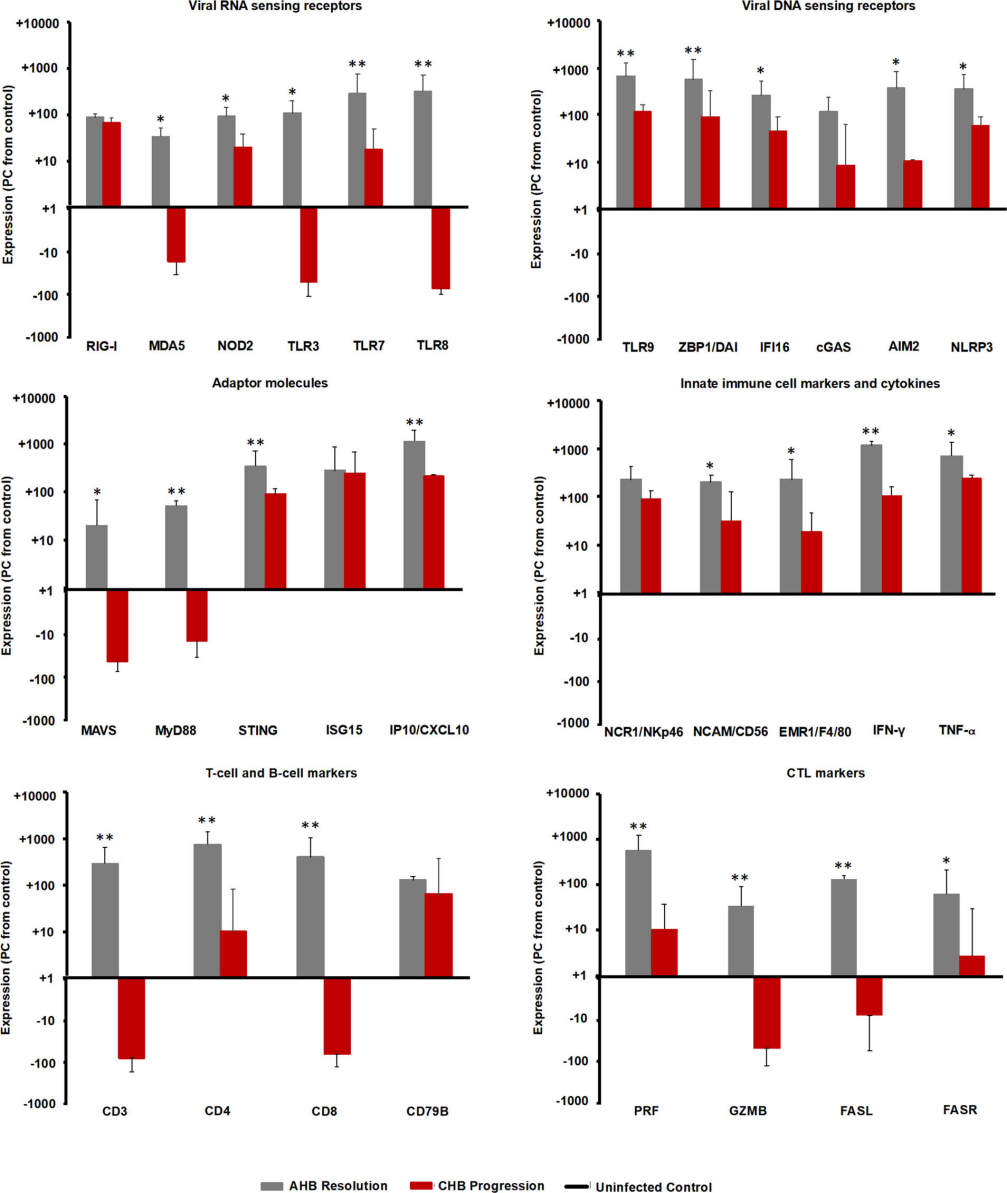
Comparison of intrahepatic expression of PRRs, adaptor molecules, ISGs, innate and adaptive immune cell markers, and cytokines during the resolved and chronic outcomes of WHV infection. Liver expression of important viral RNA and DNA sensing receptors, adaptor molecules, ISGs, innate and adaptive immune cell markers, and cytokines in ten woodchucks each with AHB resolution or CHB progression was compared to their expression in WHV-uninfected controls (i.e., in the liver obtained from the ten woodchucks with normal or delayed AHB resolution prior to WHV inoculation). The marker expression was averaged for each setting (i.e., peak AHB, CHB, and uninfected control). The percentage increase or decrease in expression relative to the uninfected control is presented. Horizontal bars represent the standard error of the mean. *P* values representing statistical significance during AHB resolution compared to CHB progression are shown as * for <0.05 and ** for <0.01. PC, percentage-change.

Since the intrahepatic kinetics of PRRs and their adaptor molecules during normal and delayed resolution were comparable and mainly differed in regard to the timepoint of peek expression, the upregulation of various PRRs in the liver of adult woodchuck during the course of acute WHV infection suggested their involvement in AHB resolution. In the setting of human CHB, *in vitro* studies have shown that the high amounts of HBV antigens interfere with the function of selected PRRs (25, 26). For determining *in vivo* differences in PRR expression that may depend on the antigenemia level present during acute and chronic WHV infection (i.e., relatively low *versus* high WHV surface and e antigen loads), the peek expression of important receptors during AHB resolution and CHB progression was compared to their baseline expression in WHV-uninfected controls (i.e., in the liver of the ten animals with AHB prior to WHV inoculation). This comparison (**Figure 10**) revealed that the expression of certain viral RNA sensors, including MDA5, TLR3, and TLR8, was upregulated in woodchucks during AHB resolution, but downregulated in animals with CHB progression (percentage-change: AHB, +33.1 to +319.9%; CHB, -14.3 to -52.4%). Other RNA sensing receptors, such as RIG-I, NOD2, and TLR7, were upregulated in both settings, but to a much lesser extent during CHB progression (percentage-change: AHB, +85.7 to +283.9%; CHB, +17.0 to +66.7%). Similarly, the expression of viral DNA sensors, including TLR9, ZBP1/DAI, IFI16, cGAS, AIM2, and NLRP3, was upregulated during AHB resolution, and also during CHB progression, although to a lesser degree (percentage-change: AHB, +116.9 to +646.1%; CHB, +8.1 to +115.7%).

The comparison of IHC results for selected PPRs in liver tissues from two woodchucks each with AHB resolution or CHB progression with two uninfected controls (**Supplementary Figure 7**) confirmed the receptor expression at the transcript level. The percentage of stained immune and non-immune cells during CHB progression was always lower than during the peak of AHB resolution (RIG-I, AHB, 21.8 to 29.0%, CHB, 4.2 to 6.1%; TLR7, AHB, 13.8 to 16.7%, CHB, 3.6 to 5.2%; TLR8, AHB, 17.3 to 34.2%, CHB, 3.2 to 3.6%; ZBP1, AHB, 10.0 to 24.0%, CHB, 1.1 to 2.2%; NLRP3, AHB, 11.4 to 26.7%, CHB, 2.4 to 2.6%).

Consistent with the above findings, the expression of MAVS and MyD88 adaptor molecules (**Figure 10**), both of which are involved in RNA receptor pathways, was downregulated during CHB progression when compared to AHB resolution (percentage-change: AHB, +20.0 to 51.5%; CHB, -10.0 to -26.0%), while the expression of the STING adaptor molecule that is utilized in DNA receptor pathways was upregulated (percentage-change: AHB, +345.9%; CHB, +88.4%). Upregulation was also noted for common ISGs, such as ISG15 and IFN-gamma-induced protein 10 (IP-10/CXCL10), but like STING, their expression was lower than during AHB resolution (percentage-change: AHB, +282.7 to +1,156.7%; CHB, +218.7 to +246.0%).

For determining if the upregulated expression of viral DNA sensing receptors and of half of the viral RNA sensing receptors tested is associated with an immune response during CHB progression, selected immune cell markers and cytokines were analyzed (**Figure 10**). Regarding innate immunity, the expression of NK-cell markers, including NCR1/NKp46 and NCAM/CD56, IFN-γ, and tumor necrosis factor-alpha (TNF-α) was upregulated during CHB progression, when compared to their expression in the uninfected control, but always lower than during AHB resolution (percentage-change: AHB, NKp46, +224.8%, NCAM, +193.0%, IFN-γ, +1,137.0%, TNF-α, +703.0%; CHB, NKp46, +89.6%, NCAM, +31.8%, IFN-γ, +106.5%, TNF-α, +246.12%). Since the expression of the macrophage marker EGF-like module-containing mucin-like hormone receptor like-1 (EMR1/F4/80) was reduced during CHB progression when compared to AHB resolution (percentage-change: AHB, +345.9%; CHB, +88.4%), this may indicate that macrophages/Kupffer cells are a less likely cell source for upregulated expression of viral RNA and DNA sensors and TNF-α. Adaptive immune cell markers, including CD3, CD4, and CD8 for T helper cells and CTLs, CD79B for B-cells, and effector molecules produced mainly by CTLs, such as PRF, GZMB, as well as FASR and FASL, displayed a reduced or even downregulated expression during CHB progression, when compared to AHB resolution and the uninfected control (percentage-change: AHB, CD3, +286.2%, CD4, +735.0%, CD8, +389.6%, CD79B, +125.9%, PRF, +556.4%, GZMB, +33.8%, FASL, +132.0%, FASR, +63.8%; CHB, CD3, -75.8%, CD4, +10.2%, CD8, -61.3%, CD79B, +62.7%, PRF, +10.7%, GZMB, -48.0%, FASL, -7.5%, FASR, +2.7%). These results could indicate an immune response during CHB progression, which is mainly mediated by NK-cells *via* IFN-γ for non-cytolytic viral control and *via* TNF-α for apoptosis and necroptosis of most likely virus-infected hepatocytes. Compared to the innate immunity, the adaptive immune response apparently was less present during CHB progression based on the reduced or downregulated expression of T-cell subset markers and cytolytic effector cytokines. This overall suggested an underlying weak innate immune response in woodchucks with CHB progression, most likely by type-I IFN production *via* activated viral DNA and selected RNA sensing receptor pathways, while the adaptive immune response was largely absent.

## Discussion

PRRs recognize viral nucleic acids and initiate an antiviral immune response *via* their downstream signaling pathways (24). Activation of PRRs further shapes the adaptive immune response against viral infections, including HBV (27–29). The antiviral properties of selected PRRs after activation by agonist molecules have been preclinically evaluated for possible treatment of patients with CHB (8, 30, 31). GS-9620, the first-in-class oral TLR7 agonist developed for the treatment of CHB generated CTL and NK-cell responses following the induction of IFN-α and ISGs in HBV-infected chimpanzees and in WHV-infected woodchucks (32, 33). APR002, another TLR7 agonist, produced elevated expression of ISGs and induced a functional cure in a subset of woodchucks, when administered together with Entecavir (ETV) (34). GS-9688, a TLR8 agonist, also mediated sustained antiviral effects in a subset of woodchucks (35). AIC649, an inactivated parapoxvirus ovis particle preparation that activates antigen-presenting cells (APCs) mainly *via* TLR9, suppressed viral replication in woodchucks as a single agent; however, the combination of AIC649 and ETV produced superior antiviral effects (31). Likewise, CpG 21798, a synthetic oligodeoxynucleotide containing CpG motifs for the activation of TLR9, modulated WHV replication and delayed viral relapse after treatment discontinuation, but only when provided together with ETV to woodchucks (36). Apart from TLR stimulators, the RIG-I agonist, SB9200, mediated antiviral immunity and direct antiviral activity *in vivo* (37, 38), and activation of the immune response by this compound before additional viral suppression with ETV resulted in pronounced antiviral effects in woodchucks (39). Besides these receptors, the activation of other PPRs during HBV infection and their contribution to viral control is less known. In the present study, the expression kinetics of receptors from various PRR families were investigated during acute, self-limited WHV infection in ten adult woodchucks.

For a better delineation of events during different stages of WHV infection, these woodchucks were separated into five animals each with normal and delayed WHV resolution, based on the individual kinetics of viremia and antigenemia. Compared to normal resolution, the detection of and the peeks in serum viremia and antigenemia lagged by approximately 4 weeks in animals with delayed resolution. Although a few PRRs appeared sporadically upregulated in the blood of 1 to 2 woodchucks, the average peripheral and intrahepatic expression of the 16 receptors tested did not increase during the initial 5/6 or 9 weeks of WHV infection in animals with normal or delayed resolution, respectively. The unchanged PRR expression was present despite the detection of WHV rc-DNA in serum during the first week after inoculation and a subsequent peak in viremia 4 to 8 weeks later. This is comparable to the acute HBV infection in three adult chimpanzees, during which a correlation between increasing HBV rc-DNA loads and upregulated expression of virus-induced host genes and immune response-related genes in the liver was not observed during the initial 6 weeks post-inoculation (40). The study on AHB resolution in chimpanzees, however, was unable to address the expression of various PRRs, except for IFI16, as most receptors were identified more recently. The peak expression of IFI16 in chimpanzee liver was observed at weeks 13/14 post-inoculation during the ongoing HBV clearance phase (40), which is similar to the peak expression of this and most other PPRs in the liver of woodchucks, especially in animals with delayed AHB resolution. Based on the intrahepatic PRR presence at the transcript level in woodchucks, this pointed to a stealth-like behavior of WHV, as described for HBV (8, 40–43). These studies showed that HBV and WHV fail to induce an innate immune response mediated by type-I IFNs and antiviral ISGs immediately after infection, which was also observed recently for five woodchucks of this study (10). Furthermore, in chimpanzees with AHB resolution, the kinetics of HBV-specific T-cell response and the expression of T-cell derived IFN-γ regulated genes demonstrated the importance of the adaptive immune response in viral control (40), as also shown in the current study and reported previously for woodchucks with AHB resolution (10, 41, 44).

The stealth-like behavior of hepadnaviruses, however, is currently being revisited due to recent *in vitro* findings, while the underlying mechanism of initial viral escape from the innate immune response still remains poorly understood. A few studies reported that DNA receptor pathways in primary human hepatocytes (PHHs) are sometimes less activatable by agonists due to a lower receptor presence, when compared to human blood-derived macrophages and woodchuck fibroblastoma cells (45, 46). This is in contrast to studies in which naked HBV DNA, covalently-closed circular DNA, and pre-genomic RNA were sensed in PHHs and human hepatoma cells by sufficient levels of cytoplasmic cGAS, nuclear IFI16, or nuclear and cytoplasmic RIG-I, respectively, leading to a pathway activation of the respective receptors (47–49). Furthermore, HBV virions are sensed by PHHs *via* TLR2 and induce a receptor-mediated expression of pro-inflammatory cytokines in these cells, but without activating IFNs and ISGs (50). In support of the latter studies, activation of an innate immune response in liver as early as 6 hours after experimental WHV infection was shown in adult woodchucks (44). Increased expression of IFN-γ and interleukin 12 was followed by an elevated transcription of several APC and NK- and NKT-cell markers 48 to 72 hours later, all of which was associated with a partial suppression of WHV replication although the infection was able to expand further and to reach a peak thereafter. Since this study did not test for changes in PRR expression, it is unknown if receptors were upregulated as well immediately after WHV infection. Considering the results of the current study, it appears that there is a lack phase of continued innate immune response activation in woodchuck liver and that WHV replication needs to reach a certain level in regard to viral DNA, RNA, and/or antigen loads in hepatocytes before PPRs become strongly upregulated and/or activated again. Interestingly, this marked PPR upregulation occurred after the induction of an anti-WHs antibody response that most likely modulated the levels of intracellular WHsAg and of circulating subviral particles and infectious virions, possibly indicating a role of WHV nucleic acids in PRR activation. Thus, differing from several *in vitro* findings, the average expression of all PRRs investigated in the current study was unchanged during the initial 5/6 to 9 weeks of WHV infection, but the expression of most receptors increased thereafter, and became maximal elevated at weeks 9 and 12/13 during normal or delayed resolution, respectively. Of note is that individual woodchucks with delayed resolution had a notable increase in the percentage of TLR8+ cells already at week 9, possibly indicating a difference at the transcript and protein level of this particular receptor and/or between the two resolution outcomes. At the transcript level, however, the kinetics of intrahepatic PRR expression were comparable between normal and delayed resolution, but receptor upregulation and peak expression were deferred by 3 to 4 weeks during the latter outcome. The peak expression of most PRRs was often higher in woodchuck with delayed than with normal resolution, while the presence of selected receptors sometimes differed at the protein level, with lower numbers of TLR8+ and NLRP3+ cells in the liver during delayed resolution. The underlying reason for this observation is unknown but may depend on the timing of the liver biopsies and/or different sensitivities of the PCR assay and IHC. The staining results for selected PRRs overall confirmed the differential kinetics of receptor expression between the two resolution outcomes. Taken together, the PRR results of the present study likely indicated an involvement and coordinated activation of several viral RNA (i.e., TLR7/8 and NLRC5) and DNA sensing receptors (i.e., TLR9, IFI16, ZBP1/DAI, AIM2, and NLRP3) in the successful viral control during WHV resolution. Other viral RNA and DNA sensors, such as RIG-I, NOD2, TLR3, cGAS, DHX9 and DHX36 did not show pronounced and/or prolonged expression increases during resolution. Peak receptor and pathway upregulation in the liver correlated with the induction and maximum of innate and adaptive immune responses (10), including NK-cell and CTL markers and cytolytic effector molecules, and with a decline in WHV replication in both resolution outcomes (10–12). The longer lasting viremic phase during delayed resolution apparently required more induction of CTL response and likely included additional NK-cell help for cytolytic and non-cytolytic control of the infection, based on a lower level of SDH secreted by killed WHV-infected hepatocytes and a more pronounced liver inflammation. PRR expression in the blood of three animals each with normal or delayed resolution, when detectable, was usually lower and showed different kinetics than in the liver, suggesting a preferential receptor presence and/or upregulation in the liver, the organ of viral replication and disease. However, since changes in PRR presence and expression but not in function were tested, direct receptor activation by WHV nucleic acids was undistinguishable from indirect receptor upregulation by type-I IFNs produced by already activated receptors, such as presumably TLR8 during delayed resolution. This differentiation may be important, since systemic IFN-α administration to woodchucks with CHB induced several of the investigated PRRs in the liver (51).

The inefficiency of the antiviral immune response present during chronic HBV infection in patients has been described extensively, especially for the adaptive arm (30, 52), but less is known regarding the innate arm. In the present study, the intrahepatic PRR expression in ten woodchucks each with AHB resolution or CHB progression was compared to those of WHV-uninfected controls. A similar comparison was performed for two animals each at the protein level for selected receptors. Both viral RNA and DNA sensors were clearly reduced during CHB progression, when compared to their peak expression and presence during AHB resolution. Furthermore, at the transcript but not at the protein level, several DNA receptors showed comparable or even higher upregulation than RNA receptors in the CHB setting. However, compared to the uninfected control, the expression of several RNA receptors, including MDA5, TLR3, and TLR8 was downregulated during CHB progression, while those of other RNA receptors, such as RIG-I, NOD2, TLR7, and of all DNA receptors, was upregulated, but to a lesser extent than during the peak of AHB resolution. This difference was also noted for RIG-I, TLR7, ZBP1/DAI, and NLRP3 at the protein level and to some degree for TLR8, with a receptor presence that was comparable between uninfected control and CHB progression. Upregulation of DNA receptors (i.e., CDS) during AHB resolution and CHB progression correlated with increased transcript levels of the adaptor molecule STING, as well as antiviral ISGs (i.e., ISG15 and IP-10/CXCL10). The observed ISG15 induction during CHB progression when compared to the uninfected control differs from the comparable levels in cultured liver biopsy samples from control individuals with mainly alcohol-induced liver disease and patients with CHB (43). Nevertheless, CDS and inflammasome upregulation during CHB progression correlated further with expression increases in IFN-γ and TNF-α most likely produced by NK-cells, as macrophage and T-cell markers and cytolytic effector molecules were reduced or downregulated when compared to the uninfected control or peak AHB resolution, all of which is consistent with previous reports on immune response in woodchucks with CHB (41, 53, 54). Overall, this indicated an underlying but weak innate immune response and a deficient adaptive immune response during CHB progression, both of which are apparently insufficient for controlling WHV infection, when compared to the strong immune response during AHB resolution observed in the current study and described recently (10). The cause of this impaired innate immune response is unknown but may involve a lack of WHV RNA recognition by several PRRs and/or subsequent activation of RNA receptor pathways, as suggested by the downregulated transcript levels of their MyD88 and MAVS adaptor molecules and the reduced expression of IRF3/7 transcription factors. In cell cultures and the HBV humanized mouse model (25, 55), it was shown that HBV infection and viral proteins affect type-I IFN production by inhibiting adaptor molecule and transcription factor functions of PRRs. Thus, it is conceivable that the prolonged presence and/or the higher levels of WHV surface and e antigens in woodchucks with CHB progression than during AHB resolution interfere more with the activation of RNA than DNA receptor pathways. This is further supported by the antiviral effects mediated by RNA receptor agonism *in vitro* (TLR3) and *in vivo* (TLR3/7/8/9 or RIG-I), with the latter often resulting in reduced or undetectable viremia and antigenemia levels, and antibody seroconversion in at least subsets of animals (31, 33, 35, 39, 56). Future studies should focus on understanding how HBV/WHV in the setting of CHB progression avoids efficient detection by PRRs and the induction of an innate immune response that is different to the receptor pathway activation and strong immune response induction during AHB resolution. The above studies could not differentiate between the PRR location in WHV-infected or uninfected hepatocytes. However, the IHC results for selected PRRs in the current study identified their presence in only hepatocytes (i.e., ZBP1/DAI) and in both hepatocytes and infiltrating immune cells (i.e., RIG-I and NLRP3 and more so TLR7 and TLR8). The subcellular localization of these receptors was either cytoplasmic (RIG-I, ZBP1/DAI and NLRP3) or endosomal (TLR7 and TLR8). This agrees with the described localization of these receptors in other studies (15, 21–23). The IHC results did not reveal a nuclear localization of RIG-I in woodchuck hepatocytes, as described in a recent *in vitro* study in which HBV pre-genomic RNA was sensed by a nucleus-specific RIG-I in human hepatoma cells (in addition to its more potent cytoplasmic counterpart) and resulted in the induction of type-III IFNs (47).

In summary, the present study investigated in great detail the previously unknown kinetics of receptors from different PRR families in liver and blood during AHB resolution in the woodchuck model of HBV, including the less explored receptors ZBP1/DAI, IFI16, AIM2, and NLRP3. Separation of woodchucks with different kinetics of AHB resolution did not show differences in PRR expression and presence during the early weeks after infection but revealed a correlation between the decline in viremia and antigenemia and the peak upregulation of these receptors in hepatocytes and infiltrating immune cells. The difference in PRR expression and presence during peak AHB resolution and CHB progression, as well as the correlation with immune response marker expression, indicated the involvement of these receptors in the control of WHV infection during resolution and the lack thereof during persistence. This observation supports the concept of PRR agonism that is currently explored for the induction of functional cure in patients with CHB. However, mirroring the situation in AHB resolution, parallel activation of multiple endosomal and cytosolic PRRs in hepatocytes and immune cells during CHB progression, and especially of viral RNA receptors, may be beneficial or required for inducing HBV cure.

## Data Availability Statement

The original contributions presented in the study are included in the article/**Supplementary Material**. Further inquiries can be directed to the corresponding author.

## Ethics Statement

The animal study was reviewed and approved by the Institutional Animal Care and Use Committee (IACUC) of Northeastern Wildlife, Inc. (Harrison, ID).

## Author Contributions

MS, BL, MM, SG, and SM contributed to the conception and design of the study. MS, BL, MM, and SM performed the experiments. MS performed the statistical analysis. MS wrote the first draft of the manuscript. All authors contributed to the article and approved the submitted version.

## Funding

MS, MM, SG, and SM were supported in part by grant R01CA166213 of the National Institutes of Health (NIH)/National Cancer Institute (NCI). The funders had no role in the design of the study, in the collection, analyses, or interpretation of data, in the writing of the manuscript, or in the decision to publish the results.

## Conflict of Interest

SM serves occasionally as a paid scientific consultant to Northeastern Wildlife, Inc. (Harris, ID, USA), the only commercial source for woodchucks within the United States, from which the animals of the current study were purchased.

The remaining authors declare that the research was conducted in the absence of any commercial or financial relationships that could be construed as a potential conflict of interest.

## Supplementary Material

Supplementary Figure 1 Peripheral RLR expression. Expression changes of RIG-I, MDA5, and LGP2 in blood with WHV rc-DNA kinetics of woodchucks during AHB with **(A)** normal resolution (n=3) and **(B)** delayed resolution (n=3). The fold-changes in receptor transcript level are plotted on the right y-axis, while serum WHV rc-DNA loads are plotted on the left y-axis. The horizontal, dotted line indicates the cutoff for positive expression (i.e., ≥2.1-fold increase from the pre-inoculation baseline). Pre, pre-inoculation; EOS, end of study; FC, fold-change.

Supplementary Figure 2 Peripheral NLR expression. Expression changes of NOD2 and NLRC5 in blood with WHV rc-DNA kinetics of woodchucks during AHB with **(A)** normal resolution (n=3) and **(B)** delayed resolution (n=3). The fold-changes in receptor transcript level are plotted on the right y-axis, while serum WHV rc-DNA loads are plotted on the left y-axis. The horizontal, dotted line indicates the cutoff for positive expression (i.e., ≥2.1-fold increase from the pre-inoculation baseline). Pre, pre-inoculation; EOS, end of study; FC, fold-change.

Supplementary Figure 3 Peripheral TLR expression. Expression changes of TLR 2/4/9 (top panels) and TLR 3/7/8 (bottom panels) in blood with WHV rc-DNA kinetics of woodchucks during AHB with **(A)** normal resolution (n=3) and **(B)** delayed resolution (n=3). The fold-changes in receptor transcript level are plotted on the right y-axis, while serum WHV rc-DNA loads are plotted on the left y-axis. The horizontal, dotted line indicates the cutoff for positive expression (i.e., ≥2.1-fold increase from the pre-inoculation baseline). Pre, pre-inoculation; EOS, end of study; FC, fold-change.

Supplementary Figure 4 Peripheral CDS expression. Expression changes of ZBP1/DAI and IFI16 in blood with WHV rc-DNA kinetics of woodchucks during AHB with **(A)** normal resolution (n=3) and **(B)** delayed resolution (n=3). The fold-changes in receptor transcript level are plotted on the right y-axis, while serum WHV rc-DNA loads are plotted on the left y-axis. The horizontal, dotted line indicates the cutoff for positive expression (i.e., ≥2.1-fold increase from the pre-inoculation baseline). Pre, pre-inoculation; EOS, end of study; FC, fold-change.

Supplementary Figure 5 Peripheral inflammasome expression. Expression changes of AIM2 and NLRP3 in blood with WHV rc-DNA kinetics of woodchucks during AHB with **(A)** normal resolution and **(B)** delayed resolution. The fold-changes in receptor transcript level are plotted on the right y-axis, while serum WHV rc-DNA loads are plotted on the left y-axis. The horizontal, dotted line indicates the cutoff for positive expression (i.e., ≥2.1-fold increase from the pre-inoculation baseline). Pre, pre-inoculation; EOS, end of study; FC, fold-change.

Supplementary Figure 6 Liver tissues from one woodchuck each during AHB with normal (M7392; top panels) or delayed resolution (M7249; bottom panels) was collected at the indicated timepoints and stained for RIG-I, TLR7, TLR8, ZBP1/DAI, and NLRP3 using cross-reactive antibodies. One representative image is shown for each timepoint and the corresponding changes at the RNA (fold-change from baseline) and protein level (percentage of positively stained immune and non-immune cells) are provided below each image.

Supplementary Figure 7 Comparison of intrahepatic presence of selected PPRs during the resolved and chronic outcomes of WHV infection. Liver tissues from two woodchucks each prior to WHV inoculation (uninfected control), during peak AHB resolution, or during CHB progression were stained for RIG-I, TLR7, TLR8, ZBP1/DAI, and NLRP3 using cross-reactive antibodies. One representative image is shown for each setting and the percentage range of positively stained immune and non-immune cells is provided below each image.
