# Supplementary figures and images for: Involvement of Innate Immune Receptors in the Resolution of Acute Hepatitis B in Woodchucks
Source: Front Immunol. 2021 Jul 22;12:713420. doi: 10.3389/fimmu.2021.713420 (PMC8340647; doi:10.3389/fimmu.2021.713420)

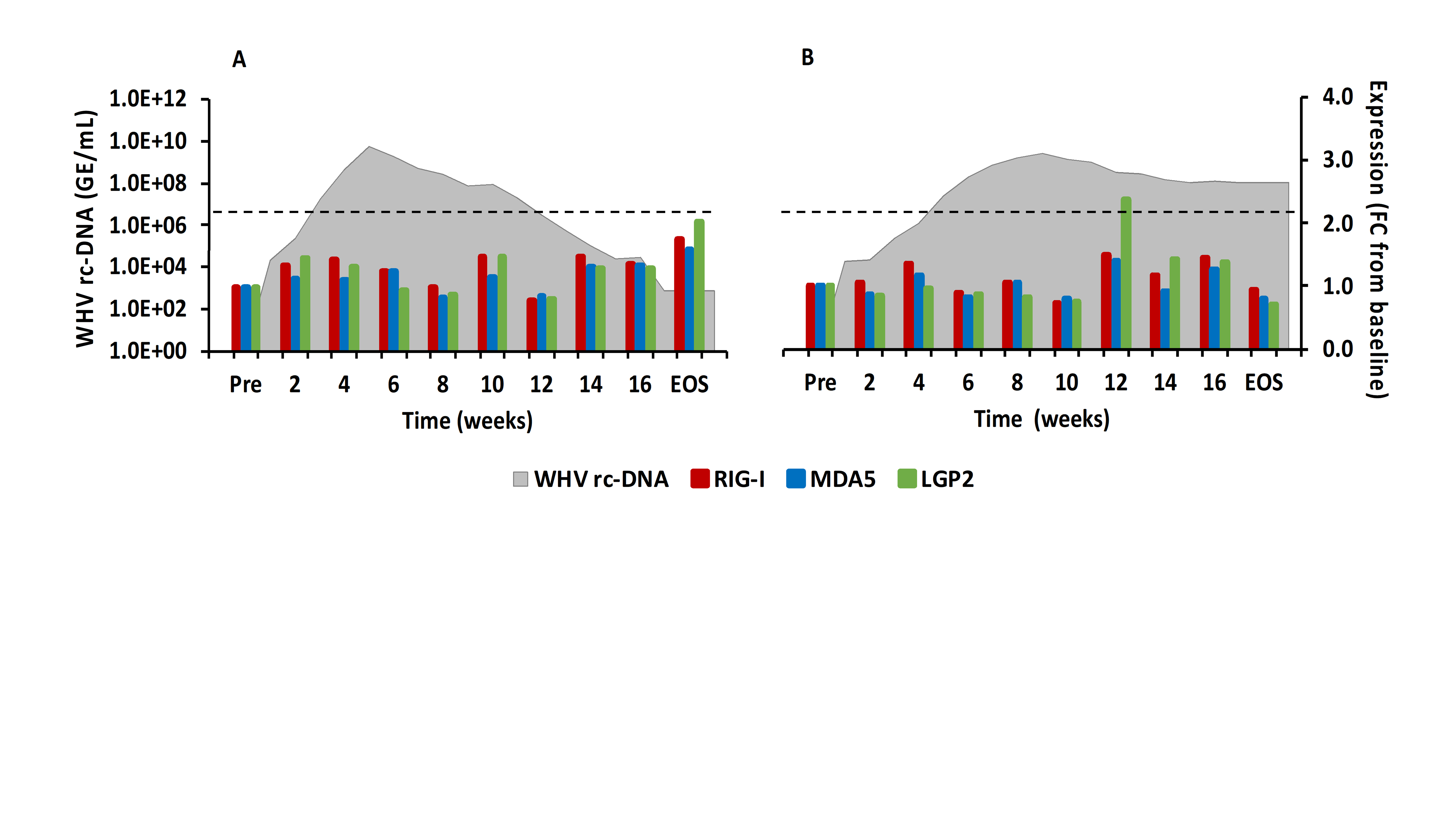

Supplement: Supplementary Figure 1 — Peripheral RLR expression. Expression changes of RIG-I, MDA5, and LGP2 in blood with WHV rc-DNA kinetics of woodchucks during AHB with (A) normal resolution (n=3) and (B) delayed resolution (n=3). The fold-changes in receptor transcript level are plotted on the right y-axis, while serum WHV rc-DNA loads are plotted on the left y-axis. The horizontal, dotted line indicates the cutoff for positive expression (i.e., ≥2.1-fold increase from the pre-inoculation baseline). Pre, pre-inoculation; EOS, end of study; FC, fold-change. [file Image_1.tif]

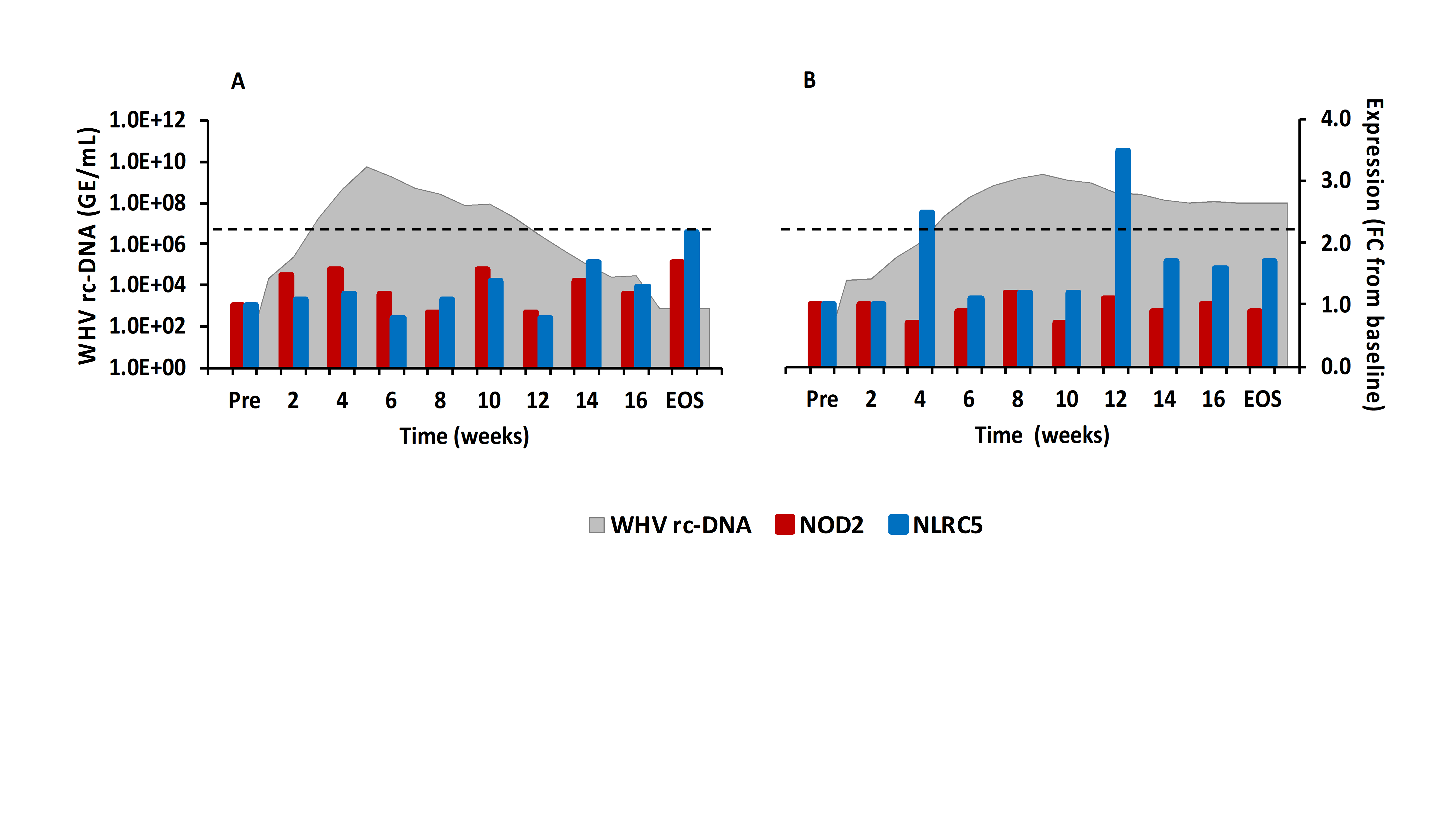

Supplement: Supplementary Figure 2 — Peripheral NLR expression. Expression changes of NOD2 and NLRC5 in blood with WHV rc-DNA kinetics of woodchucks during AHB with (A) normal resolution (n=3) and (B) delayed resolution (n=3). The fold-changes in receptor transcript level are plotted on the right y-axis, while serum WHV rc-DNA loads are plotted on the left y-axis. The horizontal, dotted line indicates the cutoff for positive expression (i.e., ≥2.1-fold increase from the pre-inoculation baseline). Pre, pre-inoculation; EOS, end of study; FC, fold-change. [file Image_2.tif]

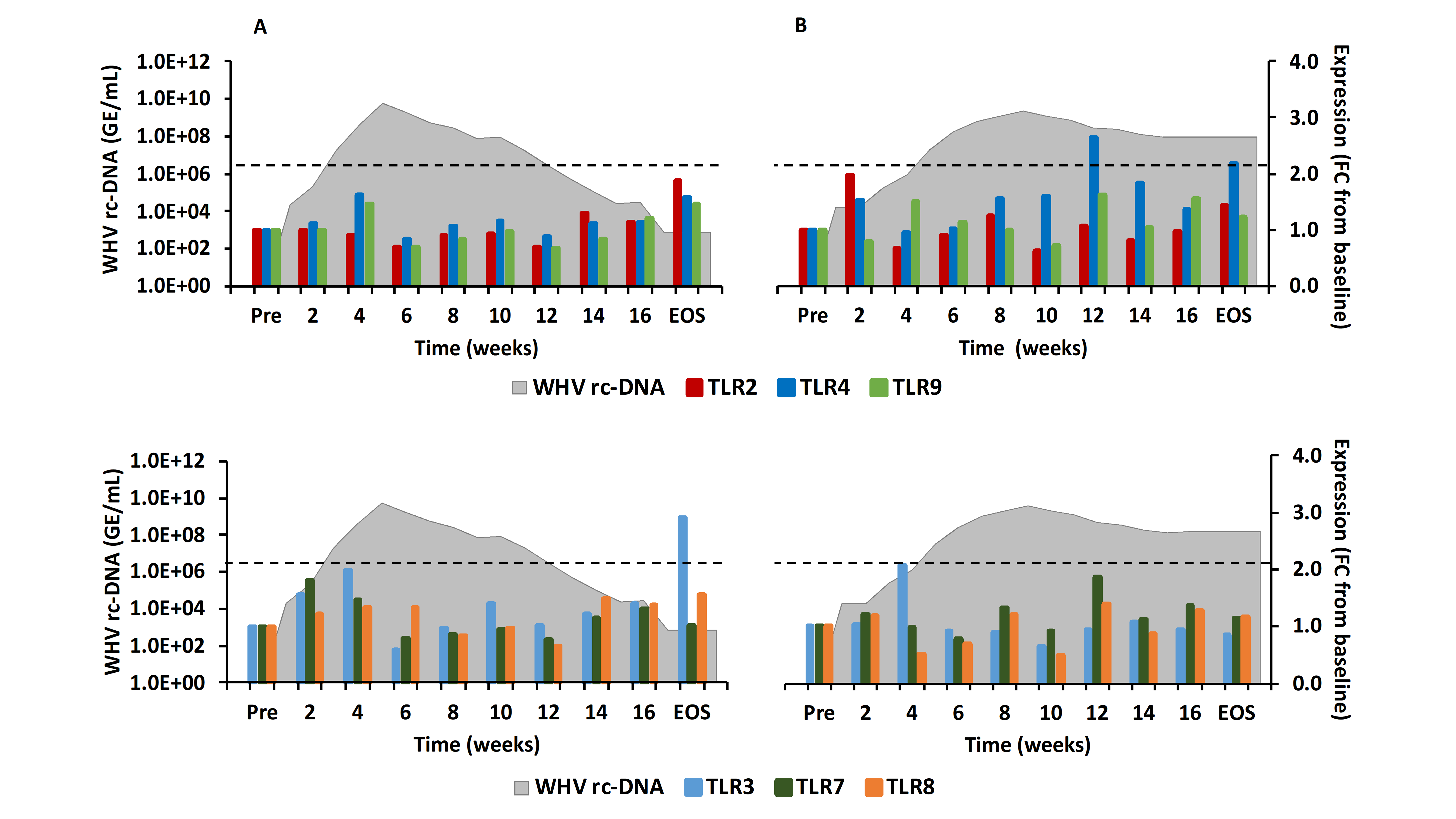

Supplement: Supplementary Figure 3 — Peripheral TLR expression. Expression changes of TLR 2/4/9 (top panels) and TLR 3/7/8 (bottom panels) in blood with WHV rc-DNA kinetics of woodchucks during AHB with (A) normal resolution (n=3) and (B) delayed resolution (n=3). The fold-changes in receptor transcript level are plotted on the right y-axis, while serum WHV rc-DNA loads are plotted on the left y-axis. The horizontal, dotted line indicates the cutoff for positive expression (i.e., ≥2.1-fold increase from the pre-inoculation baseline). Pre, pre-inoculation; EOS, end of study; FC, fold-change. [file Image_3.tif]

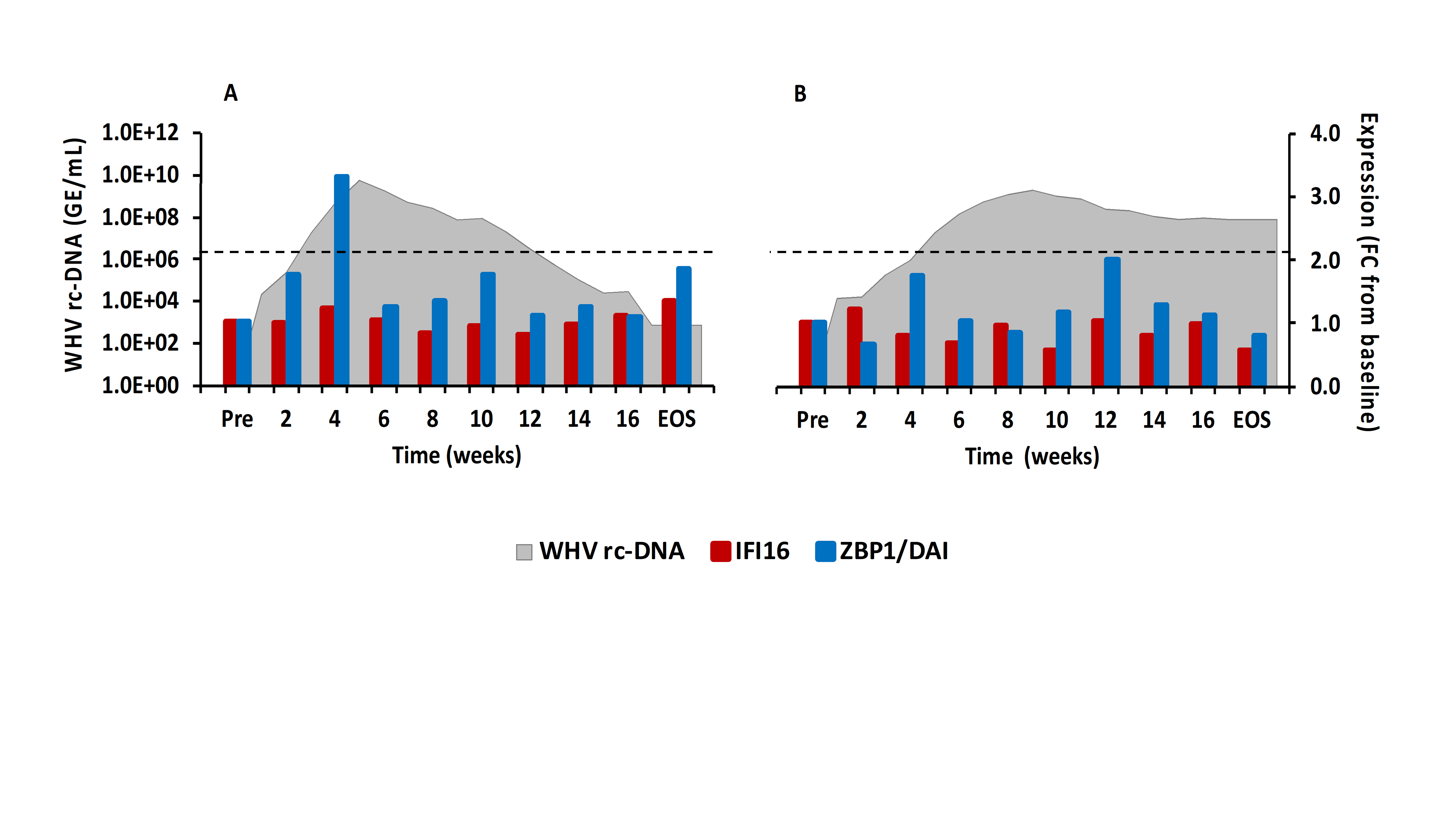

Supplement: Supplementary Figure 4 — Peripheral CDS expression. Expression changes of ZBP1/DAI and IFI16 in blood with WHV rc-DNA kinetics of woodchucks during AHB with (A) normal resolution (n=3) and (B) delayed resolution (n=3). The fold-changes in receptor transcript level are plotted on the right y-axis, while serum WHV rc-DNA loads are plotted on the left y-axis. The horizontal, dotted line indicates the cutoff for positive expression (i.e., ≥2.1-fold increase from the pre-inoculation baseline). Pre, pre-inoculation; EOS, end of study; FC, fold-change. [file Image_4.tif]

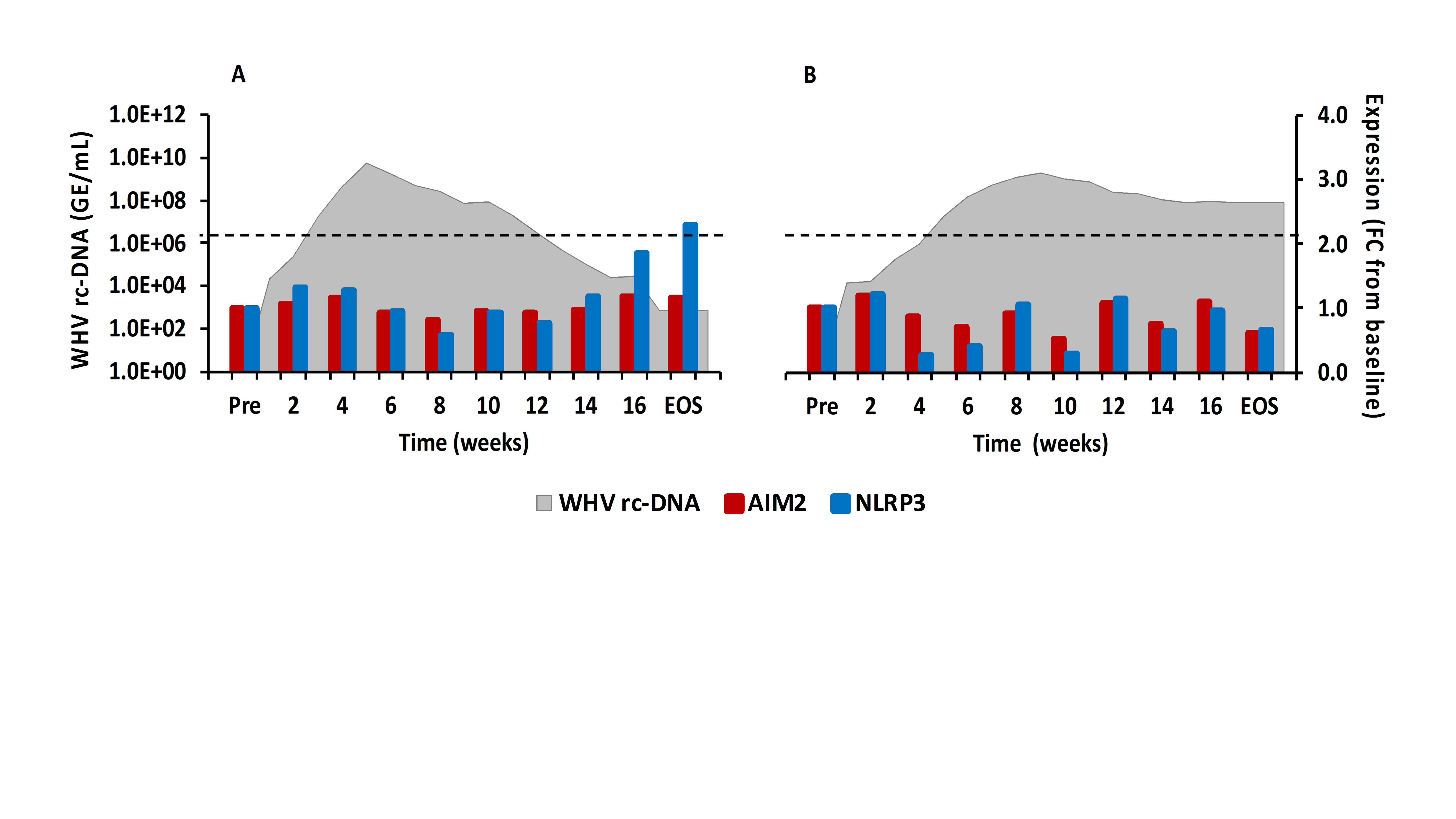

Supplement: Supplementary Figure 5 — Peripheral inflammasome expression. Expression changes of AIM2 and NLRP3 in blood with WHV rc-DNA kinetics of woodchucks during AHB with (A) normal resolution and (B) delayed resolution. The fold-changes in receptor transcript level are plotted on the right y-axis, while serum WHV rc-DNA loads are plotted on the left y-axis. The horizontal, dotted line indicates the cutoff for positive expression (i.e., ≥2.1-fold increase from the pre-inoculation baseline). Pre, pre-inoculation; EOS, end of study; FC, fold-change. [file Image_5.tif]

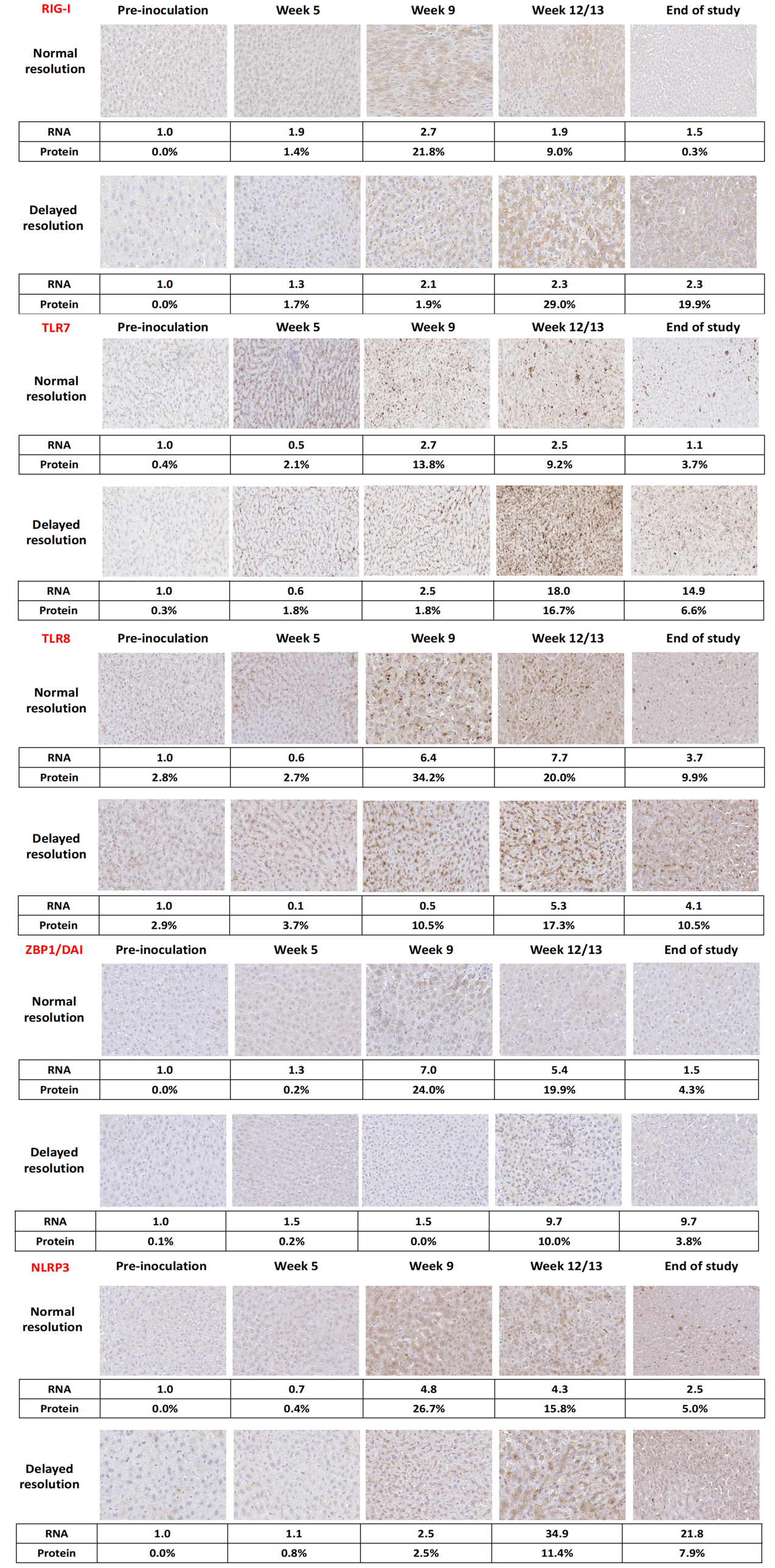

Supplement: Supplementary Figure 6 — Liver tissues from one woodchuck each during AHB with normal (M7392; top panels) or delayed resolution (M7249; bottom panels) was collected at the indicated timepoints and stained for RIG-I, TLR7, TLR8, ZBP1/DAI, and NLRP3 using cross-reactive antibodies. One representative image is shown for each timepoint and the corresponding changes at the RNA (fold-change from baseline) and protein level (percentage of positively stained immune and non-immune cells) are provided below each image. [file Image_6.jpg]

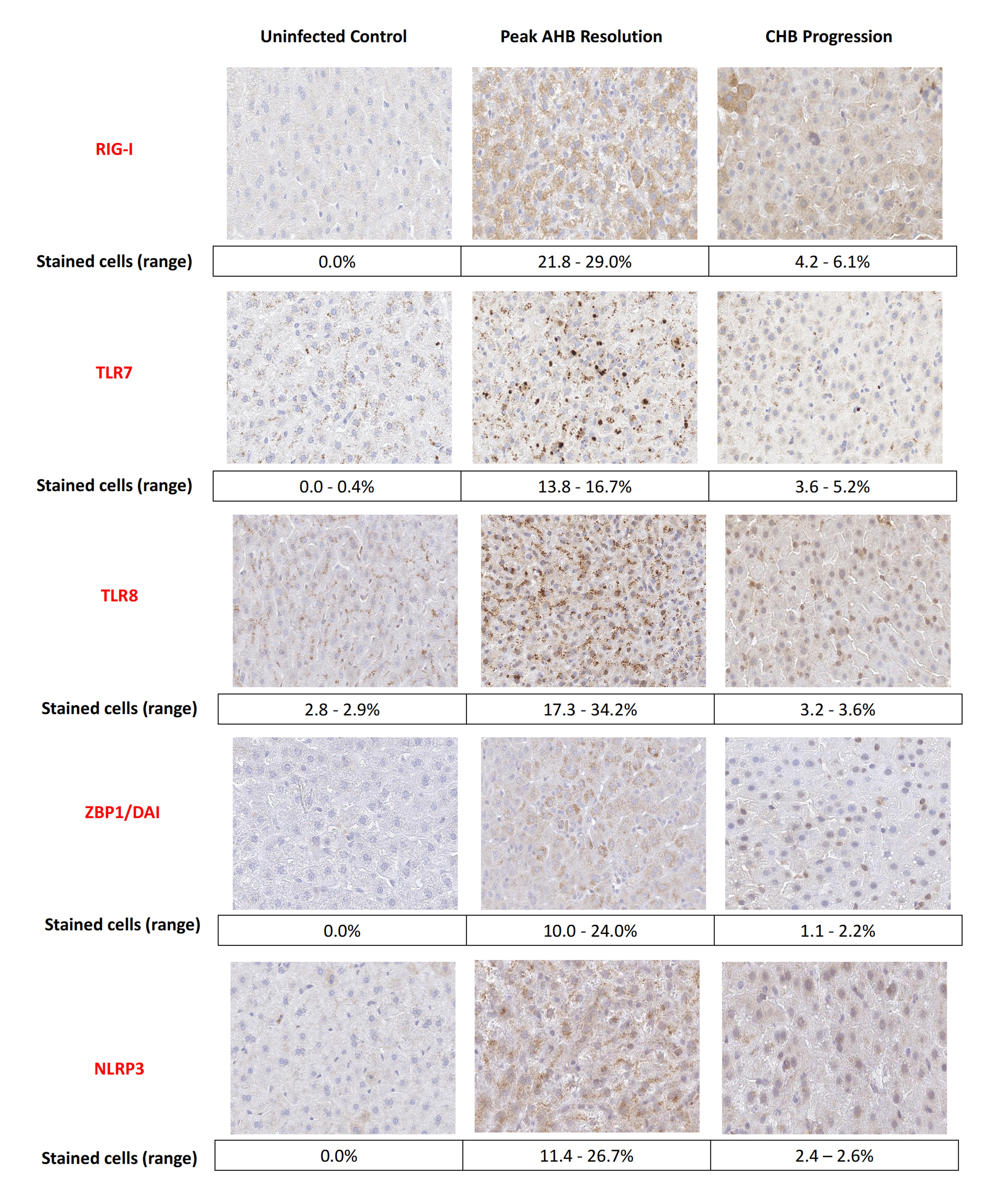

Supplement: Supplementary Figure 7 — Comparison of intrahepatic presence of selected PPRs during the resolved and chronic outcomes of WHV infection. Liver tissues from two woodchucks each prior to WHV inoculation (uninfected control), during peak AHB resolution, or during CHB progression were stained for RIG-I, TLR7, TLR8, ZBP1/DAI, and NLRP3 using cross-reactive antibodies. One representative image is shown for each setting and the percentage range of positively stained immune and non-immune cells is provided below each image. [file Image_7.jpg]
