# Supplementary material for: Involvement of Innate Immune Receptors in the Resolution of Acute Hepatitis B in Woodchucks
Source: Front Immunol. 2021 Jul 22;12:713420. doi: 10.3389/fimmu.2021.713420 (PMC8340647; doi:10.3389/fimmu.2021.713420)
Supplement: Supplementary file 8 [file Table_1.docx]

Supplementary Material

**Supplementary Table 1. Innate and adaptive immune response markers analyzed in woodchuck samples.**

| Marker Group | Genes |
| --- | --- |
| RIG-I like receptors (RLR) | RIG-I = Retinoic acid-inducible gene-I |
|  | MDA5 = Melanoma differentiation-associated protein 5 |
|  | LGP2 = Laboratory of genetics and physiology 2 |
| NOD-like receptors (NLR) | NOD2 = Nucleotide-binding oligomerization domain containing protein 2 |
|  | NLRC5 = NOD-like receptor family CARD domain containing 5 |
| Toll-like receptors (TLR) | TLR2 = Toll-like receptor 2 |
|  | TLR3 = Toll-like receptor 3 |
|  | TLR4 = Toll-like receptor 4 |
|  | TLR7 = Toll-like receptor 7 |
|  | TLR8 = Toll-like receptor 8 |
|  | TLR9 = Toll-like receptor 9 |
| Cytosolic DNA sensors (CDS) | ZBP1/DAI = Z-DNA-binding protein 1 or DNA-dependent activator of interferon regulatory factors |
|  | IFI16 = Interferon-gamma inducible protein 16 |
|  | cGAS = Cyclic GMP-AMP synthase |
|  | DHX9 = DExH-box helicase 9 |
|  | DHX36 = DEAH-box helicase 36 |
| Inflammasomes | AIM2 = Absent in melanoma 2 |
|  | NLRP3 = NOD-like receptor protein 3 or NOD-like pyrin domain containing 3 |
| Adaptor molecules | MyD88 = Myeloid differentiation primary response protein 88 |
|  | MAVS = Mitochondrial antiviral signaling protein |
|  | STING = Stimulator of interferon genes |
|  | TBK1 = TANK-binding kinase 1 |
|  | ASC = Apoptosis-associated spike-like protein coding CARD |
| Transcription factors | IRF3 = Interferon-regulating factor 3 |
|  | IRF7 = Interferon-regulating factor 7 |
| Cytokines | IFN-γ = Interferon-gamma |
|  | TNF-α = Tumor necrosis factor alpha |
| Interferon-stimulated genes | ISG15 = Interferon-stimulated gene 15 |
|  | IP-10/CXCL10 = Interferon-gamma stimulated protein 10 |
| NK-cells | NCR1/NKp46 = Natural cytotoxicity triggering receptor 1 |
|  | NCAM/CD56 = Neural cell adhesion molecule |
| T-cells | CD3 = Cluster of differentiation 3 |
|  | CD4 = Cluster of differentiation 4 |
|  | CD8 = Cluster of differentiation 8 |
| B-cells | CD79B = Cluster of differentiation 79B |
| Macrophages | EMR1/F4/80 = EGF-like module-containing mucin-like hormone receptor like-1 |
| CTLs | PRF = Perforin |
|  | GZMB = Granzyme B or cytotoxic T-lymphocyte-associated serine esterase |
|  | FASL = *F*S-7-associated surface antigen ligand |
|  | FASR = *F*S-7-associated surface antigen receptor |
