# Supplementary material for: Involvement of Innate Immune Receptors in the Resolution of Acute Hepatitis B in Woodchucks
Source: Front Immunol. 2021 Jul 22;12:713420. doi: 10.3389/fimmu.2021.713420 (PMC8340647; doi:10.3389/fimmu.2021.713420)
Supplement: Supplementary file 9 [file Table_2.docx]

Supplementary Material

**Supplementary Table 2. Oligonucleotides used for analysis of innate and adaptive immune response markers in woodchuck samples.**

| Gene | Primers and Probe | Sequence |
| --- | --- | --- |
| RIG-I | F | 5’- ACTTTGGATGGTGCCTGAAA -3’ |
|  | R | 5’- AGTGGGATACGGCGTAGAA -3’ |
|  | P | 5’- TTTCGTTGTAGGACTGGGTTGGTCC -3’ |
| MDA5 | F | 5’- GGCCAGGACATGGAGAATAA -3’ |
|  | R | 5’- CTGCCCATGTTGCTGTTATG -3’ |
|  | P | 5’- AGTTTGGCAGAAGGAAGTGTCAGCT -3’ |
| LGP2 | F | 5’- TCATCCCTGCAATGTTCTGACTCAA -3’ |
|  | R | 5’- TGGACCAAGGCTTATTCCCAGAGAAA -3’ |
|  | P | 5’- GCAGTAGAGTCCACAACAGCCATCAT -3’ |
| NOD2 | F | 5’- CTGTACGGCTTATGCCATCTT -3’ |
|  | R | 5’- ACATATCTGTGGTGGTCTTTGG -3’ |
|  | P | 5’- TCATGGATGGTGTCCAGATGCCAC -3’ |
| NLRC5 | F | 5’- ATGTCTGTGCCTCACCAATCATGT -3’ |
|  | R | 5’- ATGTGGCACTTACATGGGACTTGT -3’ |
|  | P | 5’- ACCCAGGCCATTGACATGGGACACAT -3’ |
| TLR2 | F | 5’- GATCTGCAGAGGTGTGTCAA-3’ |
|  | R | 5’- CCAGATGGCTTAGGGAAGAAA -3’ |
|  | P | 5’- CGGGCTCTGATGCTGAAGTCCAAT -3’ |
| TLR3 | F | 5’- GACCAGGTGTCTCTAAAGTTCC -3’ |
|  | R | 5’- CAGGTACAATCAAACGGGTTAAAG -3’ |
|  | P | 5’- CAGTTGAAAGAATGTGTTTGGGCCAGT -3’ |
| TLR4 | F | 5’- AAGCACCAACAGCCTCTAC -3’ |
|  | R | 5’- GTTCTCGGCACTCTTCTGTT -3’ |
|  | P | 5’- ATGGAGCTGGAACAGCCAGCTTAG -3’ |
| TLR7 | F | 5’- GGCACATTCTGAAGAGAGTTACTG -3’ |
|  | R | 5’- TGATCTAAGTGGAAATTGCCCTCG -3’ |
|  | P | 5’- TGTTCCATTTCCTTGCACACCTTGTGA -3’ |
| TLR8 | F | 5’- CAATGGCAATGGCCAAGTATTTA -3’ |
|  | R | 5’- CAGTTCACGGAGAGCATTGT -3’ |
|  | P | 5’ - TCCAAGTATTTGACCTGAGGCACAGC -3’ |
| TLR9 | F | 5’- CGCCTCTGTTTGGATGAGA -3’ |
|  | R | 5’- CAGCCACAGAGATGCTGTA -3’ |
|  | P | 5’- CTCCTGGACTTGCTTCGGCTTCTC -3’ |
| ZBP1/DAI | F | 5’- CCCAGATGTGTAGATGAGGAAC -3’ |
|  | R | 5’- AAACAGGGACGTAGCCAAA -3’ |
|  | P | 5’- CCCAGTGTGAATCCTCCCATGTCA -3’ |
| IFI16 | F | 5’- CCGAAGATCCATCAGGCAAA -3’ |
|  | R | 5’- CTACTGGATGATGCTGGAGATG -3’ |
|  | P | 5’- TCATCCAGAGGAATCCAGGGTCCT -3’ |
| cGAS | F | 5’- GGAGTCCTGCTGTAACACTTG -3’ |
|  | R | 5’- TTGCTCCAAGCCAGTTATTGA -3’ |
|  | P | 5’- TGGCCTGCTAACACACAGAAAGGT -3’ |
| DHX9 | F | 5’- CGGATCACAACAGGAGCTTTA -3’ |
|  | R | 5’- GACTGTGCTGCCAATTTCTTATT -3’ |
|  | P | 5’- TATCAAGCAGCTGGGCAGAAGGAT -3’ |
| DHX36 | F | 5’- TGGAAACAGCACTGGATACC -3’ |
|  | R | 5’- GGAGCCACTGAAGGATGATT -3’ |
|  | P | 5’- CTCCAGAGTCGGTTGCCAAGGAAA -3’ |
| AIM2 | F | 5’- CACTGATGAGGAACTGGATAGG -3’ |
|  | R | 5’- TGGCTAACTCTGTCCTGTTTG -3’ |
|  | P | 5’- CACGATTGCCAAGAGCAAACTGCA -3’ |
| NLRP3 | F | 5’- CGATCAACAGGAGAGACCTTTAT -3’ |
|  | R | 5’- CTCCTCTTCGATGCTGTCTTG -3’ |
|  | P | 5’- ATAACGCTCATGTTTCCGGTCCCA -3’ |
| MyD88 | F | 5’- CATCTTCCACCTCACTTTCTCTAC -3’ |
|  | R | 5’- CCAAGAGAGCCAGAGCAATAC -3’ |
|  | P | 5’- AGCTGTGTTCAAACCACT -3’ |
| MAVS | F | 5’- CTTGGCTTCTGTAGAGGAGATTG -3’ |
|  | R | 5’- GGCAGTAAGCTGGTAGTTGTT -3’ |
|  | P | 5’- ACTGCCATCTGCTCTAGTGATGCA -3’ |
| STING | F | 5’- CTATGAGCTTCTGGAGAATGGG -3’ |
|  | R | 5’- CTCTGCCATCCTGTGACATG -3’ |
|  | P | 5’- CTGTGTCCTGGAGTATGCCACCC -3’ |
| TBK1 | F | 5’- CTTATCTATGAAGGACGACGCTTA -3’ |
|  | R | 5’- CCCGGCTTACAACGAAGATAG -3’ |
| ASC | F | 5’- GGATATGACCACATTCGTAGGG -3’ |
|  | R | 5’- GGTCACAGTCTTCGAGTATCTT -3’ |
| IRF3 | F | 5’- TAGGCAGCTTAGTACACGTTTC -3’ |
|  | R | 5’- GACACTATCTGGACAGCCAATAA -3’ |
|  | P | 5’- TAGCCCATCACTCCTCTGTCTGTCA -3’ |
| IRF7 | F | 5’- ACCTTCACTTGCTGAGTTATCC -3’ |
|  | R | 5’- CTTGGCTGCTACTCTTCTTACTT -3’ |
|  | P | 5’- TGACCTTGGTTTACACTG -3’ |
| IFN-γ | F | 5’- ATCCAAAGGAGCATGGACAC -3’ |
|  | R | 5’- TGAACTTGAGACACCTTTAGGAA -3’ |
|  | P | 5’- CAACAGCAGTACCAATAAGCTGCAGGA -3’ |
| TNF-α | F | 5’- CCTGCAAACGGGCTATACCTT -3’ |
|  | R | 5’- GTGTGGGTGAGGAGCACGTA -3’ |
|  | P | 5’- CAGCCTTGGCCCTTGAAGAGGACCT -3’ |
| ISG15 | F | 5’- CTGTTCTGGCTGAGCTTCG -3’ |
|  | R | 5’- GCAGGTTCAGAAACACAGTGC -3’ |
|  | P | 5’- GGGAGTATGGACTCACCCCT -3’ |
| IP-10/ CXCL10 | F | 5’- AAAAAGAGCGGGGAGAAGAG -3’ |
|  | R | 5’- GGAGCCCTTTTAGACCTTTCAT -3’ |
|  | P | 5’- TCCAGAATCTAAAGCCATCAAGA -3’ |
| NCR1/NKp46 | F | 5’- CTACAGATGCTTTGGCTCCTA -3’ |
|  | R | 5’- TGCAAGACTGCTGTTCCC -3’ |
| NCAM/CD56 | F | 5’- GCAGGAGATGCCAAAGATAA -3’ |
|  | R | 5’- TCATCGATATTGGCGTTGTAG -3’ |
| EMR1/F4/80 | F | 5’- AGTGCTTTCCTGCTTTCTCATA -3’ |
|  | R | 5’- GTGGAATCTTGCATCTCCATAG -3’ |
|  | P | 5’- AGAAGGGAAATCAGACCAGGCTCC -3’ |
| CD3 | F | 5’- CGGAGTTCGCCAGTCAAGA -3’ |
|  | R | 5’- TTGGTGGTTTCCTTGAAGACG -3’ |
|  | P | 5’ - CTTCAGACAAGCAGACTCTGTTGCCCAA - 3’ |
| CD4 | F | 5’- AGGTCTCAAAGCCCGAGAAGA -3’ |
|  | R | 5’- GTAGGCACTGCCACATCCCT -3’ |
|  | P | 5’ - ATTCGGGTGCCAAACCCCAAGG - 3’ |
| CD8 | F | 5’-TGGACTTCGCCTGTGATATCTACA -3’ |
|  | R | 5’- GTTTCCGGTGGTGACAGATGA -3’ |
|  | P | 5’ - TGCGCGGTCCTTCTGTTGTCACTG - 3’ |
| CD79B | F | 5’- ACCCTCCTCATCATCCTCTT -3’ |
|  | R | 5’- CAATGTCCAGGCCCTCATAG -3’ |
|  | P | 5’- ATCGTGCCCATCTTCCTGTTGCT-3’ |
| PRF | F | 5’ – CCACCGAGCCTGACTACCTC -3’ |
|  | R | 5’ – CCAGCTCCACGGACCGGA -3’ |
|  | P | 5’ - CATCCACAACTACGGCA |
| GZMB | F | 5’- TGACATCATGTTATTGGAGCTAG -3’ |
|  | R | 5’- CCCAGGCTTCACCTTGTCC -3’ |
|  | P | 5’- TGCAGCCTATCAAGCTGCCCAGG -3’ |
| FASR | F | 5’- GTGATGGAGGACATGGCTTAG -3’ |
|  | R | 5’- TCACAGAGAGCAGTTTCACAATA -3’ |
| FASL | F | 5’- ATCCCCAGGACCTGGTGC -3’ |
|  | R | 5’- GGCCCACATCTGGCCA -3’ |
|  | P | 5’- TGGAGGGCAAGATGATG -3’ |
| 18S rRNA | F | 5’- GTAACCCGTTGAACCCCATT -3’ |
|  | R | 5’- GGGACTTAATCAACGCAAGC -3’ |
|  | P | 5’- GCAATTATTCCCCATGAACG -3’ |

F: forward primer; R: reverse primer; P: probe.
