# Supplementary material for: Involvement of Innate Immune Receptors in the Resolution of Acute Hepatitis B in Woodchucks
Source: Front Immunol. 2021 Jul 22;12:713420. doi: 10.3389/fimmu.2021.713420 (PMC8340647; doi:10.3389/fimmu.2021.713420)
Supplement: Supplementary file 11 [file Table_4.docx]

Supplementary Material

**Supplementary Table 4. Relative expression of PRRs, adaptor molecules, and transcription factors analyzed in woodchuck liver (normalized to 18S rRNA expression).**

NA, liver biopsy was unavailable; ND, not determined due to insufficient RNA amounts.
